# Dynamic Radiative Cooling: Mechanisms, Strategies, and Applications for Smart Thermal Management

**DOI:** 10.1007/s40820-025-01981-0

**Published:** 2026-01-12

**Authors:** Yan Dong, Boxi Tian, Cunhai Wang, Guoliang Zhang, Fengjiao Hua, Weifeng Meng, Chunzhe Li, Yuying Yan, Ziming Cheng, Fuqiang Wang

**Affiliations:** 1 Department of Thermal Energy and Power Engineering, Yantai University, Yantai, 264000 People’s Republic of China; 2 School of Energy and Environmental Engineering, University of Science and Technology Beijing, Beijing, 100083 People’s Republic of China; 3 School of Energy Science and Engineering, Harbin Institute of Technology, Harbin, 150001 People’s Republic of China; 4 Faculty of Engineering, University of Nottingham, Nottingham, Nottingham, NG7 2RD UK

**Keywords:** Dynamic radiative cooling, Solar energy, Radiative transfer, Radiative regulation, Thermal management

## Abstract

As an emerging thermal management strategy, dynamic radiative cooling (DRC) technology enables dynamic modulation of spectral radiation properties under varying environmental conditions through the directional design of material spectral characteristics. However, a comprehensive review of the basic physical mechanisms of radiative heat transfer in DRC materials and various design principles involved in dynamic radiative thermal regulation is still lacking. This review systematically summarizes recent advances in this field, spanning from fundamental physical principles to intrinsic molecular and electronic mechanisms, and further to representative material systems and multi-band regulation strategies, highlighting the interdisciplinary research achievements and technological innovations. This work outlines the core mechanisms governing the regulation of different spectral bands during radiative heat transfer processes. Then, the main categories of DRC materials are systematically reviewed, including actively responsive structures, passively responsive structures, and multi-stimuli-responsive materials. Furthermore, the challenges faced by current DRC technology and future development trends are summarized and discussed, providing valuable reference and guidance for further research in this field. Although DRC technologies still face significant challenges in material stability, manufacturing processes, and system integration, the continuous advances in related areas and multifunctional materials are expected to broaden the application prospects of DRC in the future.

## Highlights

- This review systematically summarizes recent advances in dynamic radiative cooling (DRC), spanning from fundamental physical principles to intrinsic molecular and electronic mechanisms, and further to representative material systems.
- This study deeply explored the innovative design of DRC technology in active response materials, passive response materials, and multi-stimuli response materials.
- The current challenges and development trends of DRC technology are comprehensively analyzed, providing reference and guidance for further research in this field.

## Graphical abstract

**Figure:**
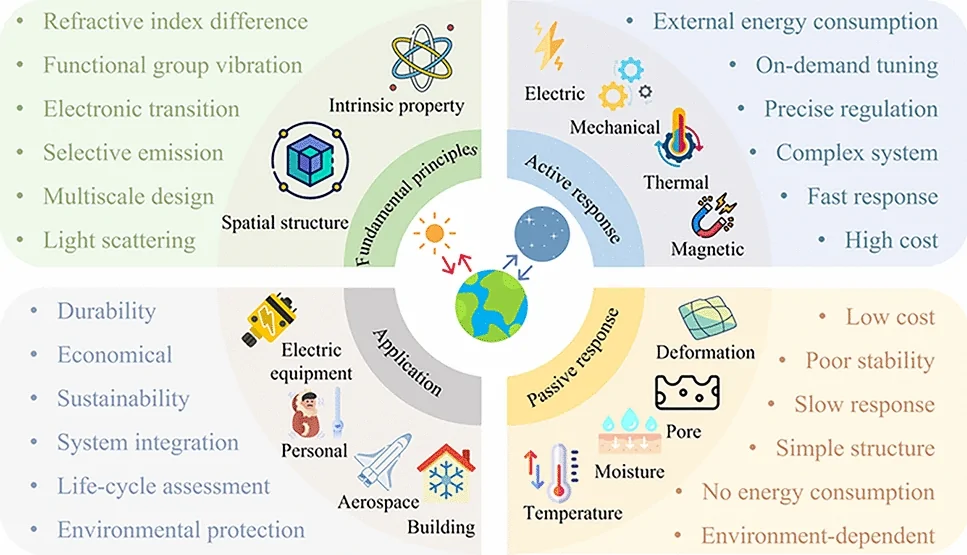

## Introduction

The over-reliance on traditional fossil fuels has not only accelerated resource depletion but also exacerbated greenhouse gas emissions, leading to severe climate change [1, 2]. Temperature regulation in living and working environments has always been a critical aspect of human development. While technological advancements over the past centuries have introduced efficient and convenient methods for heating and cooling (such as gas heating and air conditioning), these energy-intensive devices have contributed significantly to the excessive consumption of fossil fuels and the associated greenhouse gas emissions [3, 4]. To address these urgent challenges, energy conservation, emission reduction, and the development of environmentally friendly technologies have become the focus in global research, resulting in the world facing unprecedented energy crisis and environmental pressure [5]. In a pathway aligned with the IEA’s scenario for achieving net-zero energy sector emissions by 2050, accelerating energy efficiency improvements can deliver over 70% of the projected decline in oil demand [6].

**Fig. 1:**
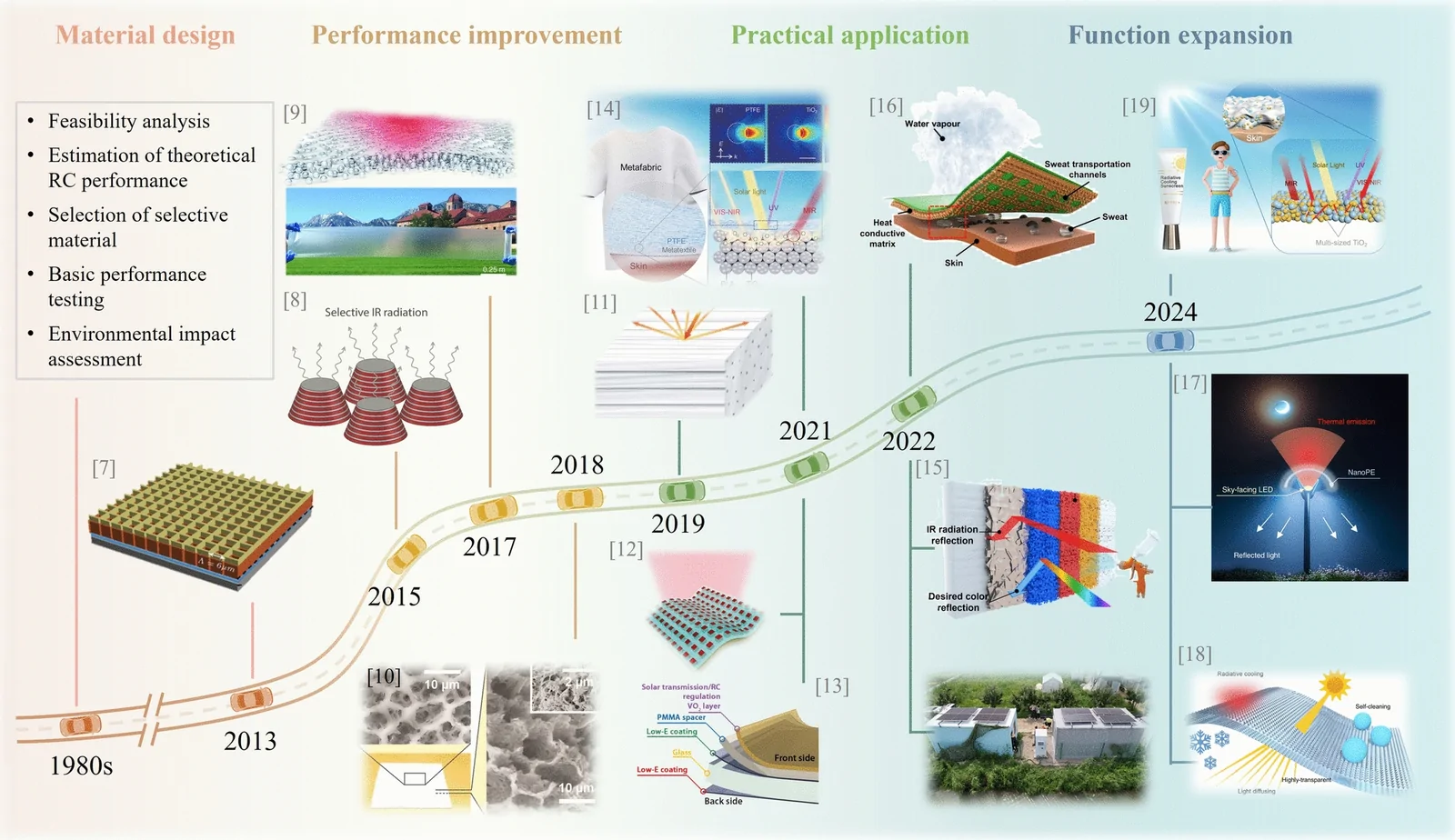
Timeline showing the development of radiative cooling technologies of four generations: materials design, performance improvement, practical application, and function expansion. “2013”: reproduced with permission [7]. Copyright 2013, American Chemical Society. “2015”: reproduced with permission [8]. Copyright 2015, John Wiley & Sons. “2017”: reproduced with permission [9]. Copyright 2017, AAAS. “2018”: reproduced with permission [10]. Copyright 2018, AAAS. “2019”: reproduced with permission [11]. Copyright 2019, AAAS. “2021”: reproduced with permission [12–14]. Copyright 2021, AAAS. Copyright 2021, AAAS. Copyright 2021, AAAS. “2022”: reproduced with permission [15, 16]. Copyright 2022, Springer Nature. Copyright 2022, National Academy of Sciences. “2024”: reproduced with permission [17–19]. Copyright 2024, American Chemical Society. Copyright 2024, Springer Nature. Copyright 2025, Springer Nature

**Fig. 2:**
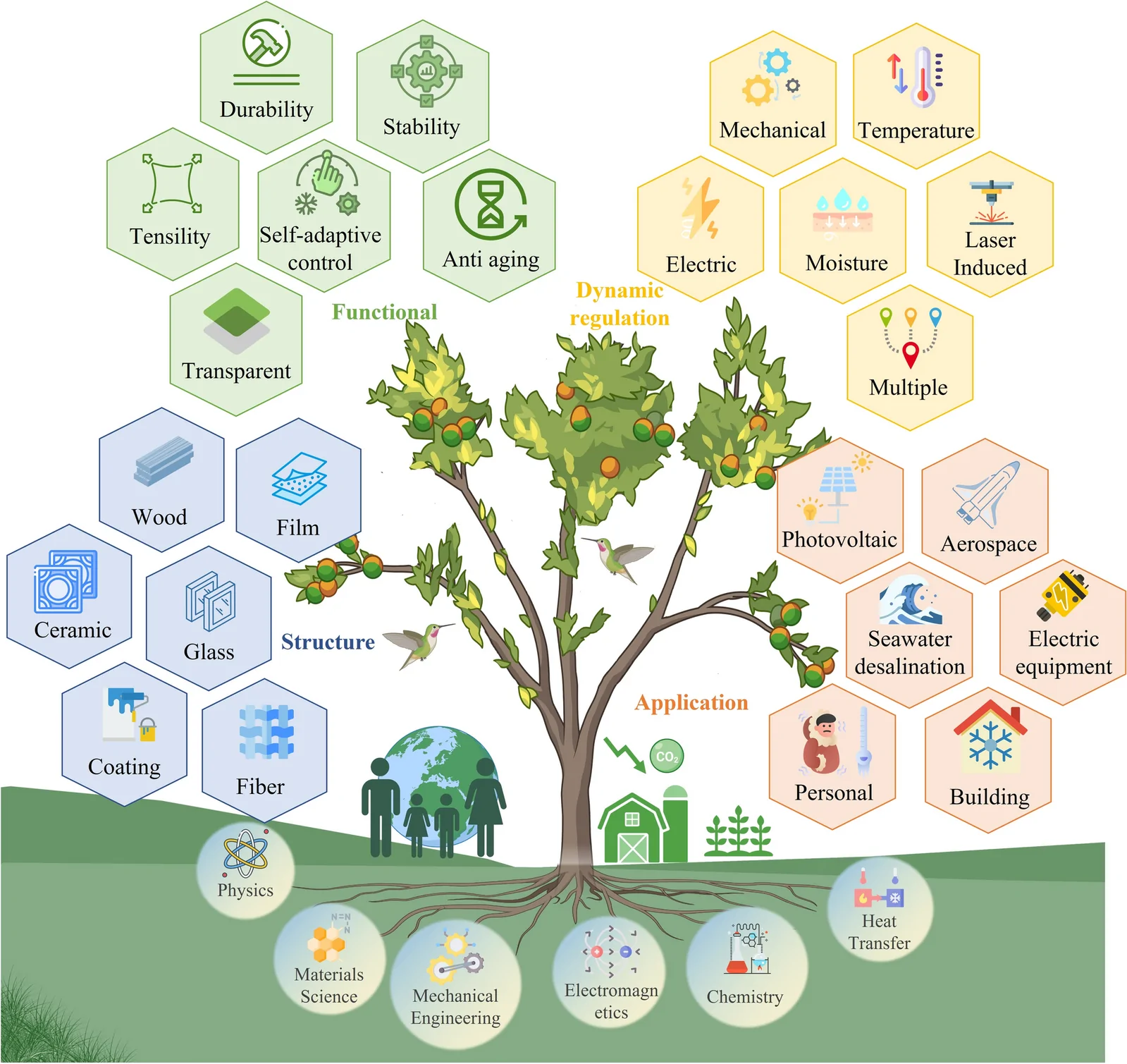
Schematic illustration of the main categories of radiative cooling materials, including structural, functional, dynamic regulation, and practical application. Taking advantage of their unique advantages in spectral selectivity and passive heat dissipation, radiative cooling materials exhibit broad development prospects across a wide range of fields. Their diversified evolution is deeply rooted in the interdisciplinary integration of physics, materials science, heat transfer, and chemistry, which collectively provide the "nutrition" for innovation and advancement in this area

Passive radiative cooling (PRC) technology has garnered increasing attention due to its distinctive capability to achieve temperature reduction without external energy input, relying solely on radiative heat transfer [20]. This passive and sustainable mechanism plays a pivotal role in energy utilization, thermal regulation, and sustainable development. At typical ambient temperatures (~ 25–30 °C), the peak wavelength of thermal emission is consistent with the wavelength range of the atmospheric transparent window (ATW, 8–13 µm). This spectral overlap enables terrestrial objects to radiate heat directly into the cold outer deep space (~ 3 K) beyond earth’s atmosphere for radiative heat exchange [21]. As shown in Fig. 1, the evolution of PRC technology can be summarized by the following time points: In 1828, Arago published the first scientific discussion on the phenomenon of PRC in a publication [22]. During the 1970s and 1980s, researchers began to explore the practical designs for PRC. With the advancement of materials science, early selective PRC materials, including polymer and metal-based coatings, laying the groundwork for efficient radiative exchange within the ATW [23]. In 1981, Ge et al. [24] calculated the cooling power of three different radiative surfaces (ideal emitter, aluminum-coated polyvinyl fluoride, and white paint with TiO_2_ particle) based on PRC technology. The calculation method of the radiation heat transfer between the radiator and the sky in the cooling system was analyzed. Since the twenty-first century, breakthroughs in nanofabrication technology and optical design theory have propelled PRC technology into a new era. In 2013, Fan et al. [7] fabricated a multilayer structure of quartz/SiC/TiO_2_/MgF_2_/silver, achieving a solar spectrum reflectance of 96.5%, an average PRC power of 105 W m^−2^, and a sub-ambient temperature reduction of 7 °C. This breakthrough research demonstrated that PRC entered the passive “daytime” radiative cooling. In 2017, Yang et al. [9] reported a mass-producible glass–polymer film capable of achieving a cooling power of 93 W m^−2^ under direct sunlight, further advancing the application and dissemination of daytime PRC technology. Nowadays, PRC has shown immense potential in various applications, including energy-efficient building design [25], personal thermal management [26, 27], preservation of food and chemical products [28], thermal regulation of electronic devices [29, 30], automotive and aerospace systems [31], and mitigation of ice melting in response to global warming [32]. The schematic diagram of main categories of PRC materials is presented in Fig. 2 to intuitively demonstrate the research foundations and evolution of current PRC technology.

Conventional PRC materials are usually static, whose spectral radiation properties remain fixed post-fabrication, posing limitations in adapting to dynamic environmental conditions such as diurnal and seasonal variations or extreme climates [33]. In the context of evolving modern energy technologies and thermal management strategies, dynamic radiative cooling (DRC) has garnered significant interest as an emerging approach that dynamically modulates radiative characteristics to achieve self-regulated across varying environmental conditions (in Fig. 2) [34]. Although the cooling capacity of DRC technology may not be as good as that of traditional PRC technology, the advantage of DRC is its compatibility with complex environments (temperature differences, humidity fluctuations, and changes in solar irradiance) [35]. To this end, multiple regulation mechanisms have been proposed, including thermal response materials [36], electrical-response materials [37], light-response materials, humidity-responsive materials [38], as well as metamaterial [39, 40]. These technologies enable materials to alter their spectral selectivity in response to external stimuli, facilitating dynamic radiative characteristic regulation.

Recently, several comprehensive reviews on dynamic radiative thermal management have been published, providing insights into the development and applications of this field [41, 42]. However, most of these reviews do not systematically analyze the fundamental physical mechanisms underlying radiative heat transfer processes or focus on the diverse design principles involved in dynamic radiative thermal management, which are critical factors influencing material selection and regulation capabilities. This review aims to systematically summarize the latest advancements in DRC technology, covering its fundamental physical principles, intrinsic regulation mechanisms, key material systems, and multi-band regulation strategies. The structure of this review is as follows: Sect. 2 provides an overview of the fundamental physical principles of radiative regulation, including radiative transfer theory and the prediction of spectral radiation properties. Section 3 delves into the intrinsic regulatory mechanisms that affect DRC regulation, analyzing the application of various material systems and control strategies in DRC. Section 4 focuses on recent advances in DRC by combining multiple regulation methods. Finally, the challenges faced by current technologies and future development trends are summarized and prospected, in order to provide reference and guidance for further research in this field.

## Fundamental Principles of Radiative Regulation

### Fundamental Physical Principles

As solar radiation traverses the Earth’s atmosphere and reaches the surface, it is attenuated due to scattering and absorption by atmospheric [43]. On a clear day, the global solar irradiance is about 1000 W m^−2^ [44]. The absorbed solar energy within the solar spectrum wavelength range (0.3–2.5 µm) is expressed as follows [45]:

1 $$ P_{{\text{solar }}} = \int_{{0.3\,\upmu {\mathrm{m}}}}^{{2.5\,\upmu {\mathrm{m}}}} \varepsilon (\theta ,\lambda )I_{{\text{solar }}} (\lambda ){\mathrm{d}}\lambda $$

where *θ* denotes the angle of incidence of solar radiation, *ε*(*θ*, *λ*) represents the spectral emittance of the object at the incidence angle *θ*, and *I*_solar_(*λ*) is the spectral intensity of solar radiation. According to the Kirchhoff’s law, under conditions of thermal equilibrium between the object and blackbody radiation, the absorptance of the object equals its emittance.

Besides solar radiation, thermal radiation is another critical parameter in process of solar heating and radiative cooling [46]. This is because all objects with a temperature greater than 0 K emit radiative energy according to their temperature and material properties, which is the fundamental physics of radiative cooling [47]. The thermal radiation power emitted by an object is a function of its thermal emittance and its temperature, described as follows:

2 $$ P_{{{\mathrm{rad}}}} (T) = \int_{0}^{2\pi } {\mathrm{d}} \Omega \cos \theta \int_{0}^{\infty } \varepsilon (\lambda ,\theta )I_{{{\mathrm{BB}}}} (T,\lambda ){\mathrm{d}}\lambda $$

where *ε*(*λ*, *θ*) is the emittance of the object at wavelength *λ* and angle* θ*, *T* is the temperature of the object, and *I*_BB_(*λ*, *T*) is radiative intensity from a blackbody at temperature *T*. *I*_BB_(*λ*, *T*) can be used to describe the spectral emissive power per unit area, per unit solid angle (Ω) for wavelength *λ* at absolute temperature *T*, as follows:

3 $$ I_{{{\mathrm{BB}}}} \;\left( {\lambda , \, T} \right){ = }\frac{{2hc^{2} }}{{\lambda^{5} }}\frac{1}{{{\mathrm{e}}^{{hc/(\lambda k_{{\mathrm{B}}} T)}} - 1}} $$

where *k* is regarded as the Boltzmann’s constant with the value of 1.3807 × 10^–23^ J K^−1^, *h* = 6.625 × 10^–34^ J Hz^−1^ is known as the Planck constant, and *c* is the velocity of light in vacuum with the value of 2.998 × 10^8^ m s^−1^. Thermal radiation had a maximum intensity at a wavelength that depended on the temperature of the substance. For example, the surface of sun is about 5800 K and is surrounded by vacuum (*n* = 1), its emission peak is close to the middle of the visible spectrum (~ 0.5 μm). In contrast, the surface of the earth in the vicinity is 290–300 K, the earth emitted thermal radiation that is mainly long wavelength infrared and invisible, and the earth’s peak emission appeared in the intermediate infrared (~ 10 μm), resulting in infrared cameras and detectors for night “vision.”

In solar heating, thermal radiation emitted by the object tends to counterbalance the absorbed solar radiation, especially at higher temperatures. Efficient solar heating requires materials with high solar absorptivity and controlled thermal emissivity. According to the energy balance equation, the net heating or cooling power *P*_net_ of an object can be expressed as follows [48]:

4 $$ P_{{{\mathrm{net}}}} (T) = P_{{{\mathrm{rad}}}} (T) - P_{{{\mathrm{sol}}}} - P_{{{\mathrm{atm}}}} (T_{{{\mathrm{atm}}}} ) - P_{{\text{non - rad}}} $$

Here *P*_ner_(*T*) represents the net power, where a negative value indicates heating power and a positive value denotes cooling power. *P*_atw_ is the absorbed atmospheric radiation power, and *P*_non-rad_ represents the non-radiative heat transfer power. The specific calculation method has been explained in detail by other researches [49].

### Interaction of Light with Objects

**Fig. 3:**
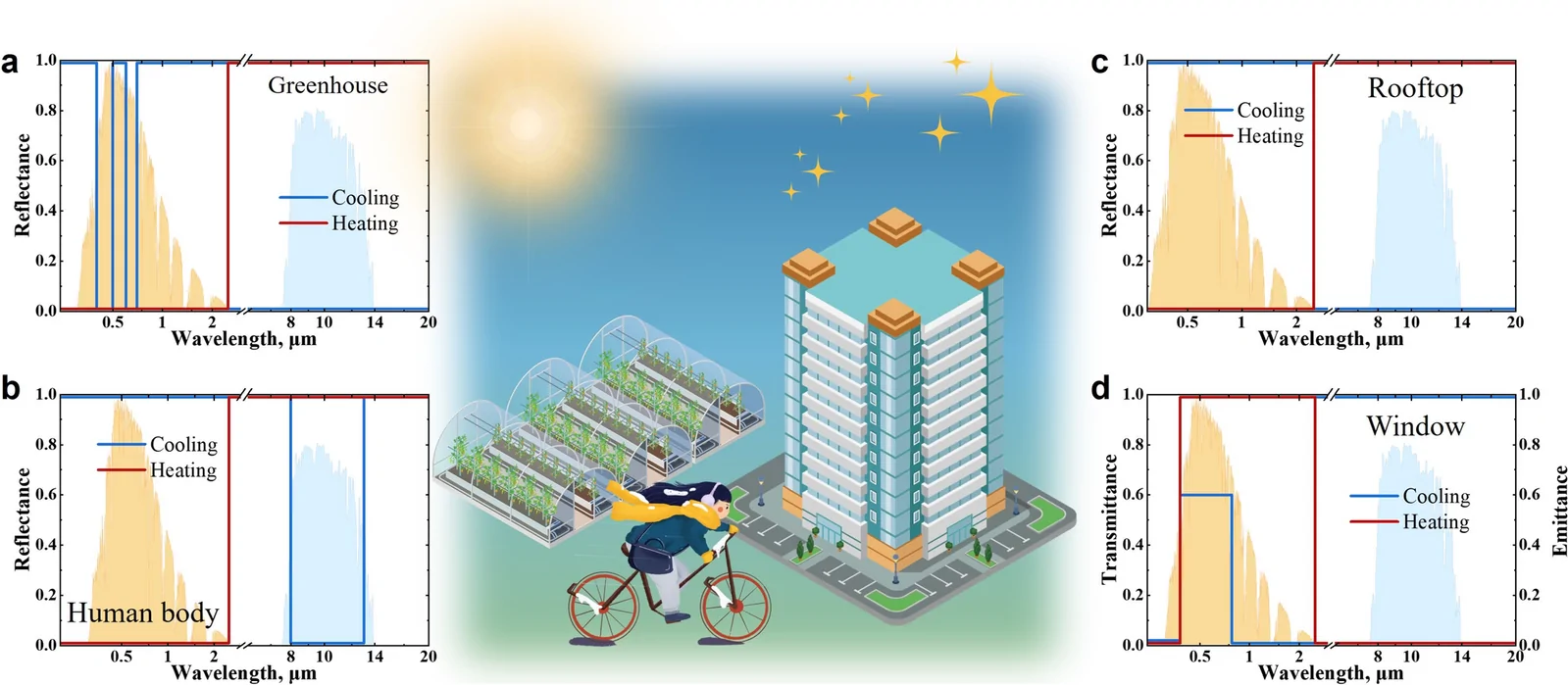
Schematic and ideal spectra of DRC to **a** greenhouse, **b** human body, **c** rooftop, and **d** window

Dynamic radiative thermal management, spanning from the solar spectrum composed of ultraviolet (UV, 0.3–0.36 µm), visible light (VIS, 0.36–0.78 µm), and near-infrared (NIR, 0.78–2.5 µm) to the broadband infrared spectrum encompassing mid-wave infrared (MWIR, 3–8 µm) and long-wave infrared (LWIR, 8–13 µm, ATW band), is importance for the directional design of spectral radiation properties [50]. As illustrated in Fig. 3, the requirements for spectral radiation properties vary significantly across different application scenarios. In spacecraft thermal protection, it is essential to modulate the surface emittance based on orientation to ensure thermal stability in space [51]. In the field of infrared camouflage, the thermal radiation properties of the object are required to match the dynamic background changes to achieve visible light (naked eye recognition) and infrared band (machine recognition) camouflage [52–54]. Information encryption requires the ability to be directionally detected by the detection equipment under dynamic conditions [55, 56]. In the field of building energy conservation, the enclosure structure needs to dynamically adjust its radiation heat transfer capacity to reduce energy consumption [57, 58].

**Fig. 4:**
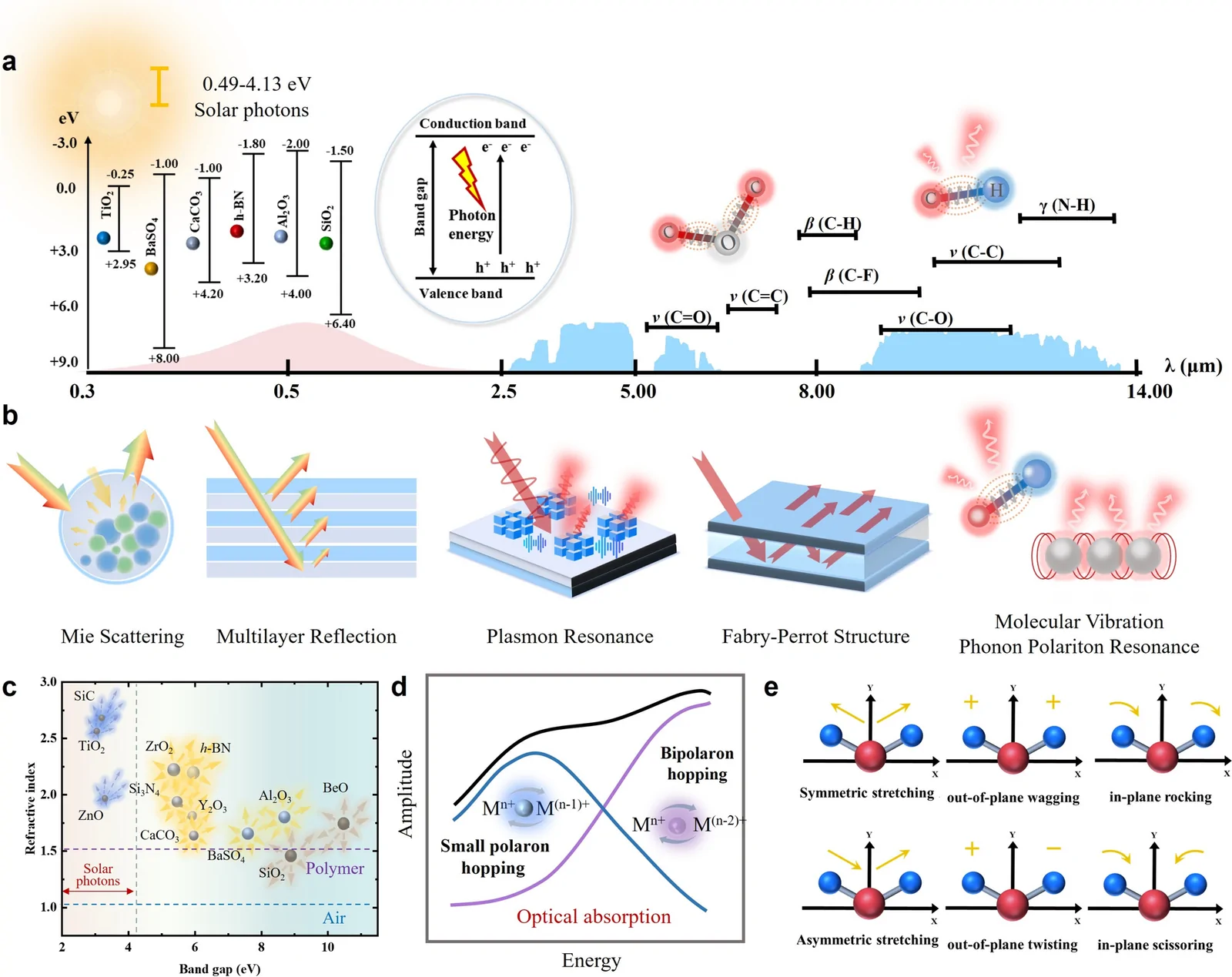
Fundamental intrinsic properties of materials. **a** The bandgap and functional groups of commonly used DRC materials in the range of 0.3–14 μm. **b** Summary of design strategies for representative DRC materials. **c** Refractive index and bandgap distribution of commonly used dielectric particles. **d** Optical density and photon energy of small polaron hopping and bipolaron hopping [81]. Copyright 2025, AIP Publishing. **e** Vibration modes of functional groups in polymers [61]. Copyright 2023, Elsevier

The focus of radiative thermal regulation is to understand the interaction between electromagnetic waves and object. Thermal radiation, an electromagnetic process driven by thermal vibrations and quantum transitions of charged particles within an object, represents a fundamental mechanism of energy transfer from the surface of an object in the form of photons [59, 60]. As illustrated in Fig. 4a, the interaction between object and light manifests microscopically as absorption and scattering, and as absorption, transmission, and reflection on a macroscopically scale, which represent the macroscopic manifestations of the interaction of light with atoms or molecules [61]. These processes collectively dictate the optical properties of materials, and the radiative properties of the material can be designed by controlling these interactions. Various materials such as photonic crystals [62], multilayered films [63], nanoparticles [64–66], porous polymers [67, 68], and other materials have been developed for the regulation of spectral radiation properties. However, their mechanisms for achieving exceptional reflection/absorption vary in principle. To elucidate the methodologies for modulating radiative characteristics in detail, this review categorizes such modulation into two distinct spectral domains: the solar spectrum and the infrared spectrum. The modulation strategies are further classified into active, passive, and multi-band coupling approaches. As shown in Fig. 4b, radiative thermal regulation materials exhibit distinct excitation transition mechanisms under varying energy intensities. In the solar band, the primary mechanisms involve electrons excitation in inorganic dielectric materials, vibrations and acceleration in the free electrons of metallic materials, and vibrational transitions in polymers. In the infrared band, the dominant mechanisms are molecular vibrations of chemical bonds or functional groups and phonon–polariton resonance within the Reststrahlen band of polar dielectrics [69]. By strategically combining these different excitation mechanisms, it is possible to achieve effective regulation of radiative characteristics over a broad wavelength range.

It is obvious from the definition that the design of *R*_solar_ and *ε*_LWIR_ plays a pivotal role in achieving the desired radiative cooling or heating performance of materials. The microscopic structure of matter typically formed by atoms, ions, or molecules bound through chemical bonds or electromagnetic interactions. The absorption of specific electromagnetic waves by matter is essentially determined by its intrinsic material properties. In the solar band, absorption is primarily driven by electron transitions within molecules, with photon energies ranging from 0.49 to 4.13 eV [70, 71]. According to the photoconductive effect, if the energy required to excite an electron from the valence band to the conduction band is less than the energy of the incident solar photons, the electrons absorb the photons, transitioning from the ground state to higher energy orbitals. Dielectric materials with larger bandgaps exhibit lower solar absorption due to their restricted electronic excitation [72, 73]. The refractive index and energy gap of semiconductors represent two fundamental physical aspects that characterize their optical and electronic properties. The energy gap determines the threshold for absorption of photons in semiconductors, and the refractive index in the semiconductor is a measure of its transparency to incident spectral radiation [74]. A correlation between these two fundamental properties has significant bearing on the band structure of semiconductors, which helps to evaluate the performance of bandgap engineering to achieve optimal absorption of broad band spectral sources. The relationship between threshold wavelength and refractive index n can be expressed as follows [75]:

5 $$ \frac{{n^{4} }}{{\lambda_{{\mathrm{e}}} }} = 77{/}\upmu {\mathrm{m}} $$

If energy gap is used as the standard, it can be expressed as [76]:

6 $$ n^{4} E_{{\mathrm{g}}} = 96\,{\mathrm{eV}} $$

The bandgap and refractive index of typical inorganic materials are summarized in Fig. 4c, including but not limited to BaSO_4_ (7.6, 1.64), Al_2_O_3_ (8.8, 1.78), TiO_2_ (3.0, 2.56), SiO_2_ (9, 1.46), CaCO_3_ (6, 1.62), h-BN (5.96, 2.2), and ZnO (3.3, 1.96) [77–79]. Taking BaSO_4_ as an example, it is often chosen as a candidate dielectric material for two reasons: (1) Its sufficiently large bandgap can minimize solar absorption, and (2) its complex crystal structure and optimal bond strength enable it to achieve high infrared emission through strong four-phonon scattering within the Reststrahlen bands [80]. The change of the intrinsic material properties will cause changes in its own spectral radiation properties. According to the work of Wen et al., for example, the dual-band regulation of amorphous cathodic electrochromic oxide MO_2_ (M represents a specific transition metal) can be achieved through the combination of small polaron hopping between M^n+^ and M^(n−1)+^ sites and bipolaron hopping between M^n+^ and M^(n−2)+^ sites, which can independently regulate the absorption in the visible and near-infrared bands (Fig. 4d) [81].

Another approach to control *R*_solar_ is through Mie scattering, which involves the scattering of incident solar light. Scattering is a phenomenon where the direction of photon propagation changes due to interactions with matter [82, 83]. Mie scattering occurs particularly when the size of the scattering medium (e.g., spheres, infinite cylinders, or other geometric shapes) is comparable to the wavelength of the incident light (*λ*), and the refractive index of the scattering medium (*n*_2_) differs from that of the surrounding medium (*n*_1_) [84]. Generally, a scatterer with a size similar to the wavelength of incident sunlight exhibits high Mie scattering efficiency (*Q*_scat_). Therefore, altering the dielectric contrast and scatterer size can effectively control the capture and diffusion of photons. By combining multiple dielectric materials with specific proportions and structures [85, 86], spatial heterogeneity in dielectric distribution can be engineered to modify the propagation path of electromagnetic waves, thereby achieving the regulation of the spectral radiative characteristics [87, 88].

The absorption in the infrared band is closely related to the vibrational transitions of chemical bonds or functional groups within a material [89]. Figure 4a shows several typical vibration frequencies of chemical bonds or functional groups. The bending and stretching of chemical bonds usually occur in the wavelength range of 400–4000 cm^−1^ (2.5–25 µm), such as C–O (1050–1310 cm^−1^, 7.6–9.5 µm) and C-H (700–900 cm^−1^, 11.1–14.3 µm) [90]. These vibrational frequencies align with the photon energy of the infrared region, leading to selective absorption or emission of specific wavelengths [91]. For example, polydimethylsiloxane (PDMS) is a widely used material with high infrared emission due to its molecular bonds, such as Si–O (1019 cm^−1^) and Si–CH_3_ (873 cm^−1^), which contribute to its a high *ε*_LWIR_ [92, 93]. Similarly, the presence of C–F (1234–1279 cm^−1^), C–H (855–976 cm^−1^), and C–H_2_ (812–840 cm^−1^) in polymers polyvinylidene fluoride (PVDF) and polytetrafluoroethylene [94, 95] makes them promising for radiative cooling. As illustrated in Fig. 4e, six common vibrational modes arise due to variations in bond length and bond angle: two stretching vibrations (symmetric in radial direction and antisymmetric in radial direction) and four bending modes (scissoring in latitudinal direction, rocking in latitudinal direction, wagging in longitudinal direction, and twisting in longitudinal direction) [96, 97]. The degree and type of vibrational modes are determined by the number of atoms and the molecular geometry. Since vibrational transitions interact with electromagnetic waves through changes in dipole moment or polarizability, stretching vibrations generally exhibit higher intensity than bending vibrations, and asymmetric vibrations are stronger than symmetric vibrations [98]. Additionally, the high infrared emission of these materials is also attributed to strong interfacial interactions. Interfacial interactions and hydrogen-bond interactions promote an inhomogeneous electric charge distribution within chemical bonds, thereby increasing the variation in dipole moment during molecular vibrations [99].

Another factor that determines *ε*_LWIR_ is the crystal structure of the material. In the infrared spectrum, the interaction between light and matter involves electron transitions and the response of free carriers [100]. Semiconductor materials can change their band structure through bandgap, doping, or defect, thereby achieving selective absorption or reflection of infrared spectrum. Vanadium dioxide (VO_2_) exemplifies this concept, exhibiting a phase transition between metallic and insulating states depending on its crystal structure. In its metallic phase, VO_2_ typically displays infrared emission, whereas in its insulating phase, it is known for high infrared transmittance, resulting in lower *ε*_LWIR_. This phase change behavior has been widely applied in smart windows and optical switching materials [101, 102]. Moreover, materials designed based on surface plasmon resonance effects hold significant potential in the infrared spectrum. Noble metal nanoparticles, such as gold and silver, exhibit strong surface plasmon resonance effects in the near-infrared range, leading to pronounced absorption or reflection peaks at specific wavelengths [103]. This design strategy is valuable in optical filters and smart windows [104]. Specific switching mechanisms and detailed principles will be discussed in the following sections, categorized by control methods.

## Dynamic Radiative Cooling Materials

### Classification Dynamic Radiative Thermal Management

At present, a variety of control methods have been developed to meet the needs of DRC, each with its own unique mechanisms and advantages. Thermal response materials, such as VO_2_ and GeSbTe, use temperature-induced phase transitions to achieve spectral regulation within specific thermal thresholds, thereby enhancing or suppressing thermal radiation [105]. Electrical tunable materials, exemplified by indium tin oxide (ITO) and AZO, use external electric field to modulate free carrier densities, enabling dynamic spectral control through plasma resonance [106]. Photo- and humidity-sensitive materials, including polymeric liquid crystal (LC) and photochromic compounds, undergo rapid molecular reconfiguration under external illumination or humidity variations, thereby altering their spectral radiation properties [107]. In addition, with the development of metamaterials and metasurface technologies, the design of DRC technology has gradually diversified, achieving the improvement of modulation efficiency and broadening of spectral tunable range [108].

The internal logic of dynamic radiative thermal management can be divided into two aspects: spatial structure and intrinsic material properties. As described in Sect. 2, by using the spatial variation of the refractive index and the variation of the electromagnetic properties of the material itself under different excitations to change the propagation path and mode of electromagnetic waves, thereby achieving adjustable reflection, absorption, and transmission of thermal radiation [109], which will be discussed in the following chapters.

### Active Response Structure

Depending on whether energy is consumed during the regulation process, dynamic radiative thermal management can be classified into active and passive response. The most prominent feature of active response is that the timing of activation being under the control of the manager, independent of environmental fluctuations, but at the cost of external energy consumption and associated low spatial efficiency. Active response refers to the radiative thermal regulation of material spectral radiation properties through external stimuli such as electric fields, mechanical, and magnetic fields. It offers advantages of rapid response times and high precision in regulation but necessitates additional energy input and complex control systems. The following sections will provide a comprehensive understanding of active response through a categorized discussion.

#### Electrical Response

Electrically responsive regulation is a dynamic radiative thermal management for real-time adjustment of optical and thermal properties by altering the internal electronic distribution or band structure of materials through applied voltage or current. This mechanism is primarily based on the electrochromic effects or electrochemical doping mechanisms, which modulate optical constants (e.g., refractive index and absorption) and thermal radiation properties by controlling free carrier concentration or ion intercalation/deintercalation. Electrochromic materials, which exhibit exceptional optical regulation capabilities under electrical stimulation (encompassing precise control over *R*_solar_ and *ε*_LWIR_, low driving voltages, and ultra-fast switching speeds), hold significant promise for thermal radiation regulation [92]. DRC technologies based on electrochromic materials are steadily advancing, with key approaches including reversible electrodeposited metals and conductive metal oxides [110, 111], conjugated conductive polymers [112, 113], and valence/state-switching metal oxides [114, 115].

**Fig. 5:**
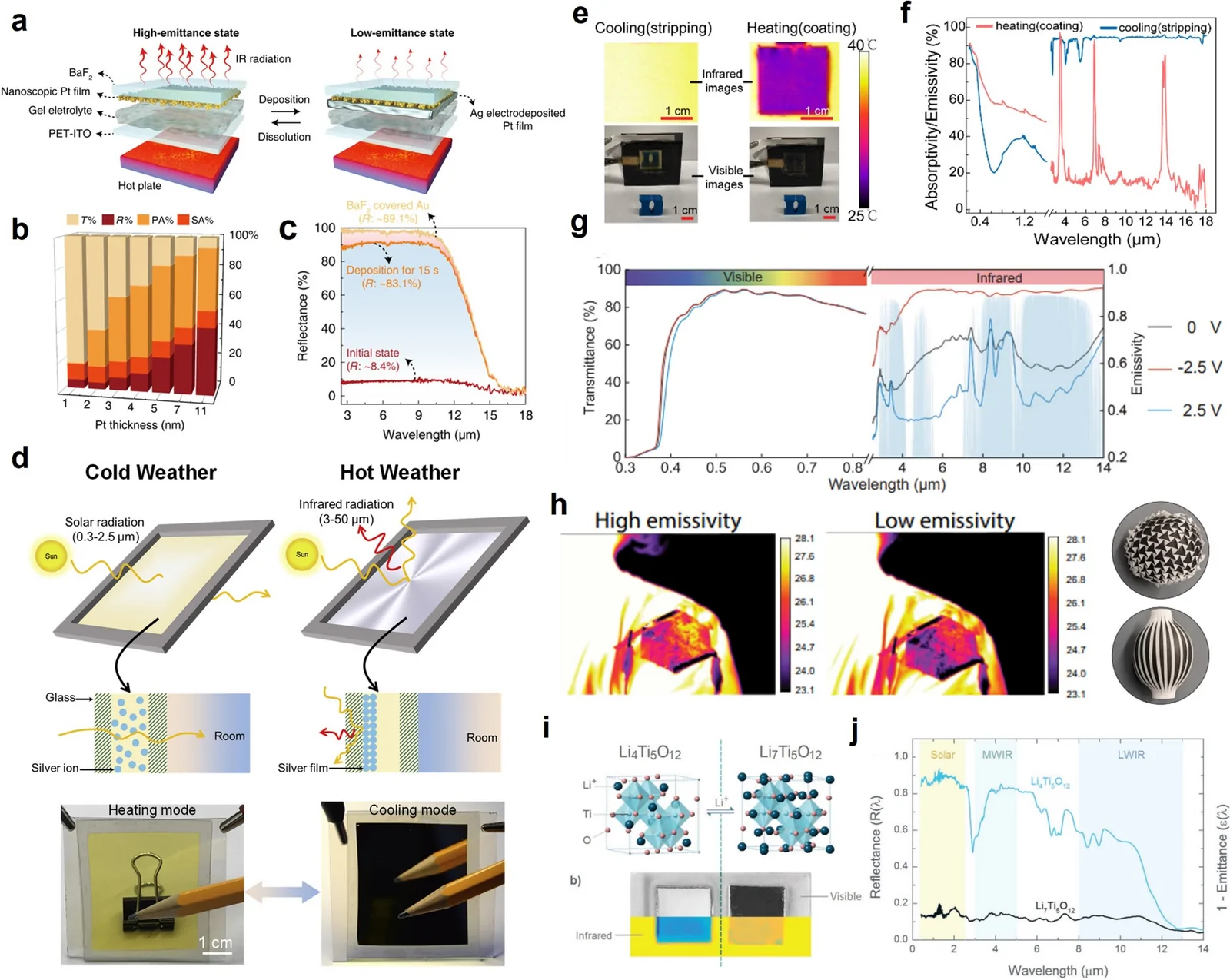
Electrical response DRC materials. **a** Schematic demonstration of the Pt film-based device and **b** the control capability of the electrochromic system under different Pt layer thicknesses [116]. Copyright 2020, AAAS. **c** Infrared reflectance spectra of the 3 nm Pt/BaF_2_ substrate before and after Ag electrodeposition (15 s) in an RSE three-electrode system [116]. Copyright 2020, AAAS. **d** Schematic demonstration and photos of the dynamic glazing panel in heating and cooling modes, respectively [118]. Copyright 2022, Elsevier. **e** Images and **f** spectra of device prepared by Hsu at the cooling and heating states [119]. Copyright 2021, ACS Publications. **g** Spectra of transparent dynamic infrared emissivity regulators at various applied voltages [120]. Copyright 2023, Springer Nature. **h** Thermal images and photos of the kirigami-enabled electrochromic wearable device [121]. Copyright 2023, National Academy of Sciences. **i** Crystal structures, photograph, and **j** spectral characteristic of the Li_4_Ti_5_O_12_- and Li_7_Ti_5_O_12_-based broadband electrochromic material [122]. Copyright 2018, John Wiley & Sons

For the reversible electrodeposited metals, color switching is achieved through controlled electrochemical metal deposition/dissolution or redox-induced optical modulation. Li et al. [116] employed the electrodeposition of silver nanoparticles to modulate *ε*_LWIR_. As shown in Fig. 5a, due to the high *ε*_LWIR_ of the nanoscale Pt film, the device exhibits a high-emissivity state when no metal is electrodeposited. Upon application of a deposition voltage, Ag is gradually electrodeposited on the surface of the nanoscale Pt film, gradually converting the infrared absorptivity and transmittance of the nanoscale Pt film to infrared reflectivity, thereby shifting the device to a low-emissivity state. By adjusting the thickness of the Pt layer, the devices achieved varying tunability (Fig. 5b). As depicted in Fig. 5c, when the Pt layer thickness is 3 nm, both the *ε*_MWIR_ and *ε*_LWIR_ increased with deposition time. A 15-s deposition significantly enhanced the broadband emittance from 0.08 to 0.83. Similarly, Sui et al. [117] designed a water-based flexible electrochromic material for building envelopes, utilizing the electrodeposition of Cu nanoparticles to regulate *ε*_LWIR._. In the absence of voltage, copper ions are dispersed in the electrolyte, resulting in high *ε*_LWIR_ due to the strong infrared absorption of the electrolyte. Upon the application of deposition voltage, copper ions are electrodeposited onto the Pt–graphene electrode surface, transforming the device into a highly reflective state and thus achieving low *ε*_LWIR_. This reversible electrodeposition exhibits excellent performance, with broadband infrared emittance spectra confirming a thermal emittance variation from 7% to 92%. Zhao et al. [118] developed a dual-mode material capable of switching between cooling and heating models. As shown in Fig. 5d, reversible electrodeposition of a silver film on transparent glass allows for the reflectance to switch between 89% and 17%. In the cooling mode, the material utilizes PRC to achieve a cooling power of 20–60 W m^−2^ under direct solar irradiance ranging from 560 to 970 W m^−2^ during summer. In the heating mode, the device permits approximately 70% solar transmittance and provides a net heating power of around 400 W m^−2^ under 540 W m^−2^ of solar irradiance. Hsu et al. [119] fabricated a flexible, ultra-broadband transparent conductive electrode with low sheet resistance (*R*_s_ = 22.4 Ω sq^−1^) and high optical transmittance (*T*_UV–VIS_ = 85.63%, *T*_NIR_ = 87.85%, and *T*_MWIR_ = 84.87%). As illustrated in Fig. 5e, the metal-based electrochromic device exhibits distinct image characteristics under different cameras. Moreover, by optimizing the electrodeposited morphology to control surface plasmon resonance, the device can switch between solar heating mode (*α*_sol_ = 0.60, *ε*_MWIR_ = 0.2) and radiative cooling mode (*α*_sol_ = 0.33 and *ε*_MWIR_ = 0.94) (Fig. 5f). Liu et al. [120] demonstrated an electrically controlled infrared emittance modulator that can independently regulate infrared emittance while maintaining high visible light transparency (84.7% transmittance in the 400–760 nm). The modulation of infrared emittance is attributed to changes in carrier concentration within the surface depletion layer of aluminum-doped zinc oxide nanocrystals. The modulator exhibits high emittance tunability (0.51 in 3–5 μm and 0.41 in 7.5–13 μm, Fig. 5g), rapid response (< 600 ms), and exceptional cycling stability (> 10^4^ cycles).

For the conjugated conductive polymers and valence/state-switching metal oxides, light absorption is modulated through reversible redox reactions. Wang et al. [113] fabricated a bioinspired film with infrared thermal radiation regulation inspired by the color-changing mechanism of chameleon. In this design, PANI and Ce^4+^ mimic the skin receptors and pigment capsules of chameleon skin, respectively, achieving an emittance variation of 0.58 in the wavelength range of 8–14 μm. Hsu et al. [121] developed a wearable, variable-emissivity device, which is an electrochromic film featuring a kirigami-enabled design and can provide stretchability and conformal deformation across various modes (Fig. 5h). The device allows programmable, personalized thermal regulation through electronic control. Each switching cycle requires less than 5.58 mJ cm^−2^ of energy input, extending the thermal comfort range by 4.9 °C, equivalent to a continuous power input of 33.9 W m^−2^. Yang et al. [122] fabricated an electrically stimulated thermochromic material composed of lithium titanium oxide integrated within a multilayer structure. As shown in Fig. 5i, voltage-induced transitions between the semiconductor and metallic phases of lithium titanium oxide enable switching between high reflection and high absorption states, with Δ*R*_sol_ and Δ*ε*_MWIR_ being 74% and 0.68, respectively (Fig. 5j).

#### Mechanical Response

Mechanically driven regulation is a dynamic approach that utilizes mechanical forces as external stimuli to alter the internal structure, microscopic arrangement, or geometric shape of materials, thereby changing its scattering efficiency to optical and thermal radiation properties [123–125]. Mechanical stimuli, such as flipping, rotation, compression, and stretching, are among the most widely employed methods for manipulating material states, which mainly including elastic materials, film materials, and Janus materials. For instance, in thin film materials, mechanical bending can modify surface morphology, thereby adjusting spectral radiation properties. Elastic materials, when subjected to mechanical forces, experience changes in molecular spacing or alignment, leading to alterations in their spectral radiation properties [126, 127]. Similarly, certain nanomaterials exhibit lattice changes under mechanical stress, which, in turn, affects their radiative properties. Moreover, mechanical deformation materials, such as wrinkled films and micro/nanostructured surfaces, undergo geometric reconfiguration in response to mechanical forces, facilitating dynamic tuning of their optical behavior. For example, the stretching of wrinkled films induces surface flattening, thereby reducing surface roughness and consequently modifying light absorption characteristics.

**Fig. 6:**
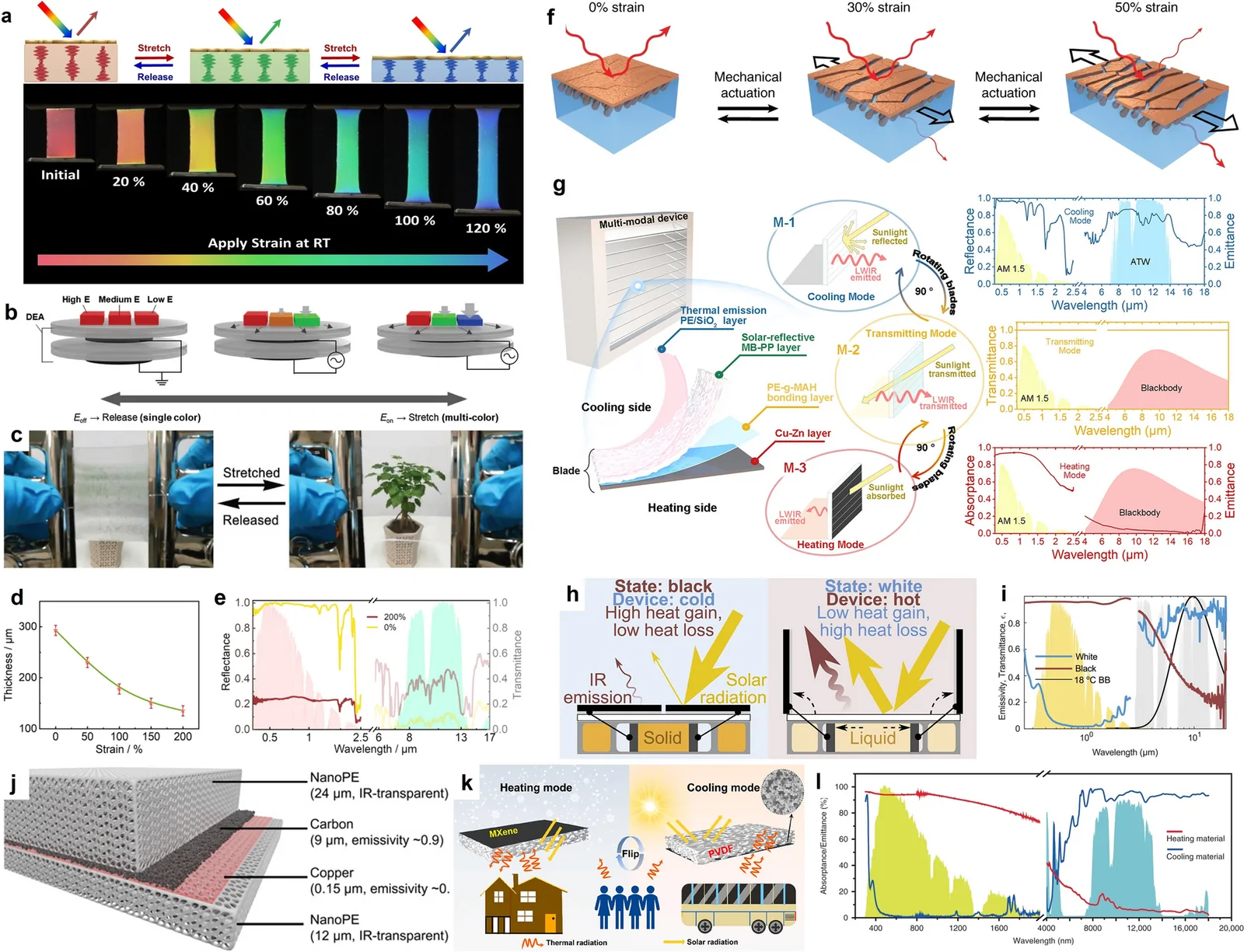
Mechanical response DRC materials. **a** Schematic illustration (top) and photographs (bottom) of the mechanochromism LC elastomer [128, 129]. Copyright 2022, John Wiley & Sons. Copyright 2025, John Wiley & Sons. **b** Invisible chameleon photonic e-skin control with multicolored change [130]. Copyright 2023, John Wiley & Sons. **c** A large size PVA/PDMS bilayer film with surface wrinkles for smart windows [131]. Copyright 2018, John Wiley & Sons. **d** Thickness and **e** spectral characteristic of the styrene ethylene butylene styrene Ag film with different strain states [134]. Copyright 2024, Royal Society of Chemistry. **f** Schematic of the mechanical actuation of the composite with different strains [135]. Copyright, 2019, Springer Nature. **g** Schematic and spectral characteristic of the Hierarchical-Morphology Metal/Polymer Heterostructure [143]. Copyright 2022, ACS Publications. **h** Schematic and **i** emittance of the two surfaces of the adaptive switch [144]. Copyright 2024, Elsevier. **j** Layered structure of the dual-mode textile [145]. Copyright 2017, AAAS. **k** Schematic of dual-mode film at heating (left) and cooling (right) mode [146]. Copyright 2023, ACS Publications. **l** Absorptance/emittance of dual-mode material prepared by Hsu [147]. Copyright 2020, Springer Nature

Feng et al. [128, 129] designed and fabricated a mechanochromic, shape-programmable, and self-healing cholesteric LC elastomer. Through mechanical stretching, the circularly polarized reflection of the LC elastomer can be dynamically and reversibly tuned across the entire visible spectrum (Fig. 6a). Choi et al. [130] introduced chiral photonic elastomers with simultaneous multicolor control. Electrical stretching of multimodular engineered chiral photonic elastomers on dielectric elastomer actuators can simultaneously achieve multicolor modification of chiral photonic elastomers (Fig. 6b). Jiang et al. [131] demonstrated ordered, crack-free surface wrinkles on a PDMS/PVA elastomer substrate through uniaxial stretching and releasing. This approach achieved a broad transmittance modulation range (from 6 to 91% in the visible spectrum, Fig. 6c) and a long switching cycle exceeding 2000 repetitions. Deng et al. [132] developed an electrically controlled polymer-dispersed LC smart window with PRC properties by incorporating mid-infrared emissive monomers into the conventional LC matrix. The PRC efficiency could be further modulated by adjusting the content of the infrared emissive component, film thickness, and microstructural morphology. Dynamic camouflage mechanisms are also observed in nature, where organisms alter their pore distribution in response to environmental stimuli. Inspired by the dynamic skin of chameleons, which achieve color change by adjusting the size and arrangement of guanine crystals [133]. Kim et al. [126] utilized LC elastomers to program pore structure distribution and size. This innovation enables pixelated color switching across a broad wavelength range from ultraviolet to near-infrared. As shown in Fig. 6d, e, Chen et al. [134] inspired by the dynamic skin of squid, developed a multilayer structure that utilizes a mechanical–optical coupling mechanism to achieve synchronous solar and thermal radiation regulation. This system achieved a maximum solar modulation rate of 0.72 and a thermal modulation rate of 0.3. As shown in Fig. 6f, Leung et al. [135] demonstrated a dynamic optical regulation system where mechanical stretching induces distributed microcracks on the film surface, exposing the substrate and resulting in a change in infrared emittance.

Efficiently integrating both cooling and heating modes within a single material presents significant challenges. Janus materials offer a promising solution to address this issue [136, 137]. The term “Janus” originates from the Roman god with two faces, symbolizing the ability to look both into the past and the future. Inspired by this duality, researchers have developed various Janus materials with distinct properties [138, 139]. Among them, the double-layer design is particularly suitable for all season thermal management due to its simpler manufacturing process [140]. The independence of the two components in Janus materials also enhances their tunability and control capabilities. Drawing inspiration from louver, some studies use the open/closed states of blade structures to regulate spectral characteristics [141, 142]. For example, Yang et al. [143] combined selective PRC materials with solar heating materials using adjustable blades. As shown in Fig. 6g, by rotating the blades, the system can switch between radiative cooling, solar heating, and natural daylighting. Similarly, as illustrated in Fig. 6h, Xiao et al. [144] utilized the thermal expansion of phase change materials (PCM) to drive the motion of blinds, achieving reversible cycling within a temperature range of less than 3 °C. When closed, the black selective absorbing blinds generate significant heat, while when opened, they reveal a white infrared emitting surface that facilitates heat dissipation (Fig. 6i). Cui et al. [145] demonstrated a Janus dual-mode textile capable of providing both passive radiative heating and cooling without external energy input. As shown in Fig. 6j, this dual-mode textile consists of a bilayer emitter embedded within a nanoPE layer. When the low-emittance layer faces outward, the textile functions as a heating surface, while reversing the orientation exposes the high emittance layer, enabling PRC. Shi et al. [146] developed a dual-mode structure by designing a hierarchical porous PVDF film modified with MXene nanosheets using an inverse phase method, which also can be flipped to adapt to dynamic cooling and heating scenarios (Fig. 6k). Hsu et al. [147] introduced a dual-mode device with electrostatically controlled thermal contact conductance. As illustrated in Fig. 6l, the cooling side employs a silver coating to reflect sunlight while using PDMS to maintain uniform emittance, thereby maximizing radiative cooling. Conversely, the heating side features a dark copper/zinc coating that absorbs solar energy and minimizes radiative loss.

#### Magnetic Response

Magnetically driven regulation utilizes external magnetic fields to alter the alignment or magnetization state of magnetic materials, thereby influencing their optical and thermal radiation properties (e.g., transmission, diffraction, polarization, and plasmonic properties). This approach is grounded in principles such as the magneto-optical effect, magnetically induced phase transitions, or magnetic field-induced microstructural changes [148]. The primary advantages of magnetic regulation lie in its non-contact and high efficiency, offering significant potential for applications in magneto-optical devices, magnetic sensors, and smart windows [149].

**Fig. 7:**
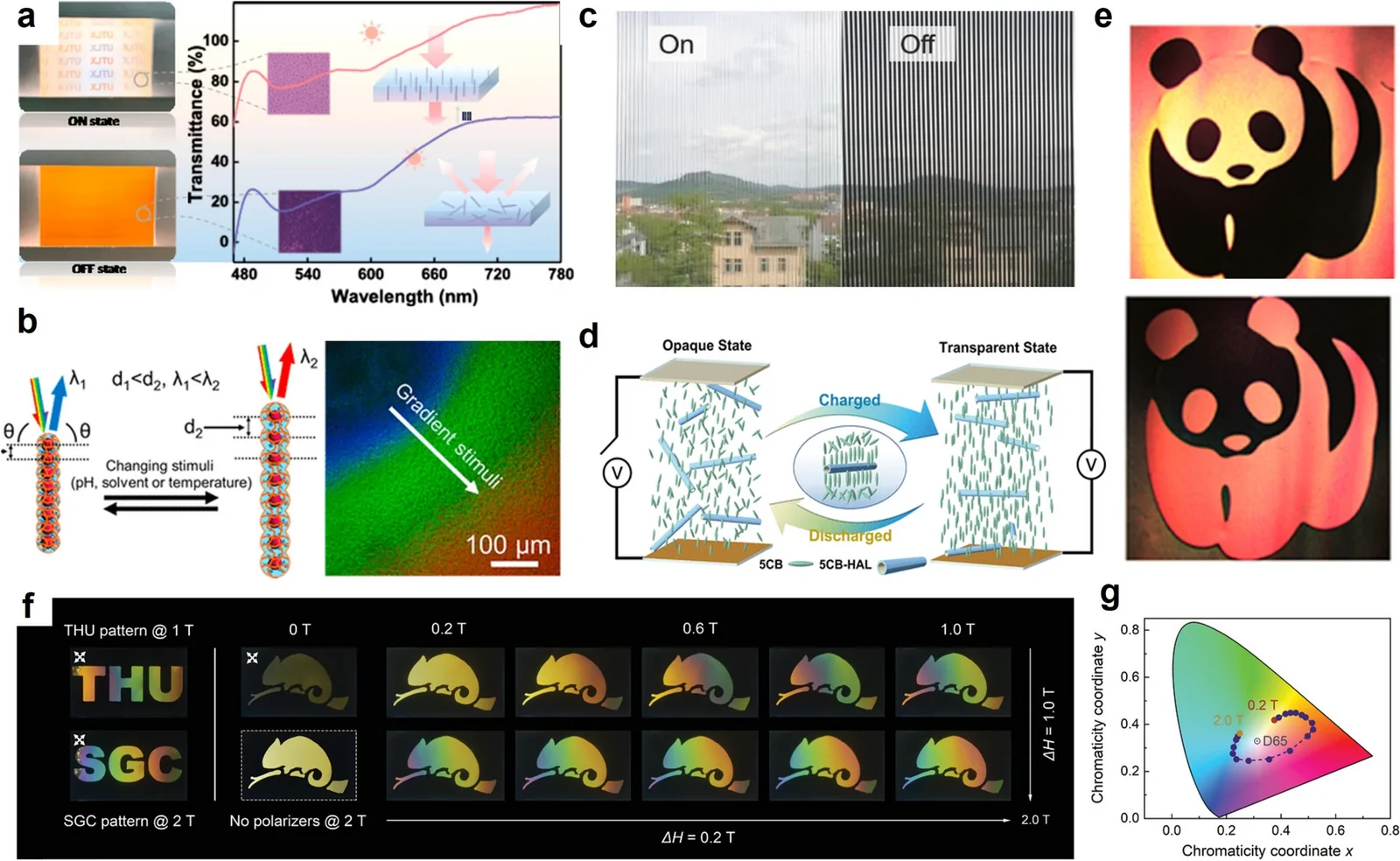
Magnetic response DRC materials. **a** Photos and transmission spectra of smart window materials with and without magnetic field [150]. Copyright 2020, ACS Publications. **b** Schematic illustration (left) and dark-field optical microscopy images (right) of pH-responsive photonic nanochains [151]. Copyright 2018, ACS Publications. **c** Photos of suspended particle device with and without charged fluid [152]. Copyright 2017, John Wiley & Sons. **d** Schematic illustration of the Nematic LC smart window [154]. Copyright 2023, American Chemical Society. **e** Polarized optical microscopy images with polarization-modulated pattern before and after shifting the direction of the transmission axis [155]. Copyright 2014, ACS Publications. **f**, **g** Color changes of color-tunable optical device with various magnetic fields and strain [156]. Copyright 2022, John Wiley & Sons

At present, the radiative thermal regulation based on magnetic response is mainly focused on the visible spectrum, and there is relatively little research on the coupling with the MWIR. As a typical magnetically controlled optical regulation system, photonic crystal structures constructed from 1D magnetic arrays on the visible light wavelength scale have garnered extensive research attention. For instance, Yang et al. [150] developed a magnetically tunable smart optical material that exhibits rapid and high-contrast optical switching. As shown in Fig. 7a, the system combines the large shape anisotropy of the 1D structure with the superparamagnetic properties of Fe_3_O_4_ nanoparticles to achieve a visible light modulation of 60%. Guan et al. [151] achieved dynamic optical regulation by altering the lattice spacing of these arrays. As shown in Fig. 7b, the reflection spectra of Fe_3_O_4_@PVP@poly(HEA-*co*-AA) PNCs vary with pH, demonstrating their sensitivity to environmental changes. Wondraczek et al. [152] demonstrated that loading circulating fluid with magnetic nanoparticles enables active shading and solar energy harvesting. As shown in Fig. 7d, the optical properties of the fluid can be remotely controlled through a particle collector-suspender device, achieving up to 45% modulation of solar radiation. Nematic LC (e.g., 5CB and 8CB) can orient carbon nanotubes in an ordered structure arrangement under the action of magnetic or electric fields [153]. Li et al. [154] constructed a highly anisotropic supramolecular LC composite by using halloysite nanotubes as a doping agent (Fig. 7e). Through the in situ grown superparamagnetic nanoparticles on the halloysite nanotube surfaces, this system achieves solar modulation of up to 75%. Yin et al. [155] designed an anti-counterfeiting label using LC composed of Fe_3_O_4_@SiO_2_ nanorods with different aspect ratios. These predesigned patterns exhibit distinct optical polarizations and produce a contrasting image under linearly polarized light (Fig. 7f). When the LC is aligned at an angle to the orthogonal polarizers, light passes through the system. In contrast, when the LC is parallel to the direction of either the linear polarizer (*P*) or the analyzer (*A*), the light is effectively blocked. Huang et al. [156] designed and fabricated a 2D inorganic LC functional material based on vermiculite. As shown in Fig. 7g, h, the material exhibits gradient color changes in response to magnetic field direction variations. The morphology, materials, key properties, and regulation ability of active response structure are presented in Table 1.

**Table 1:**
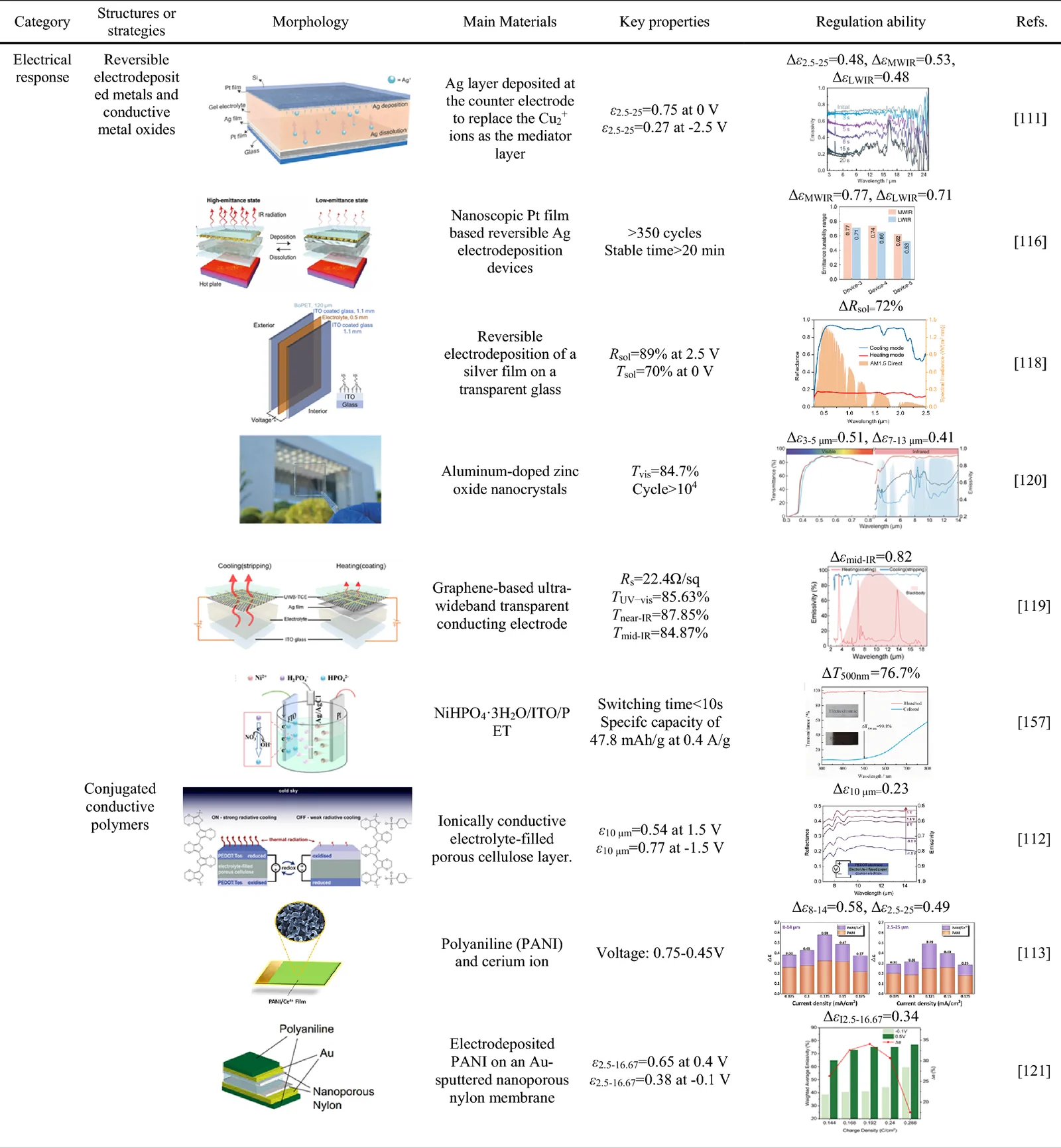

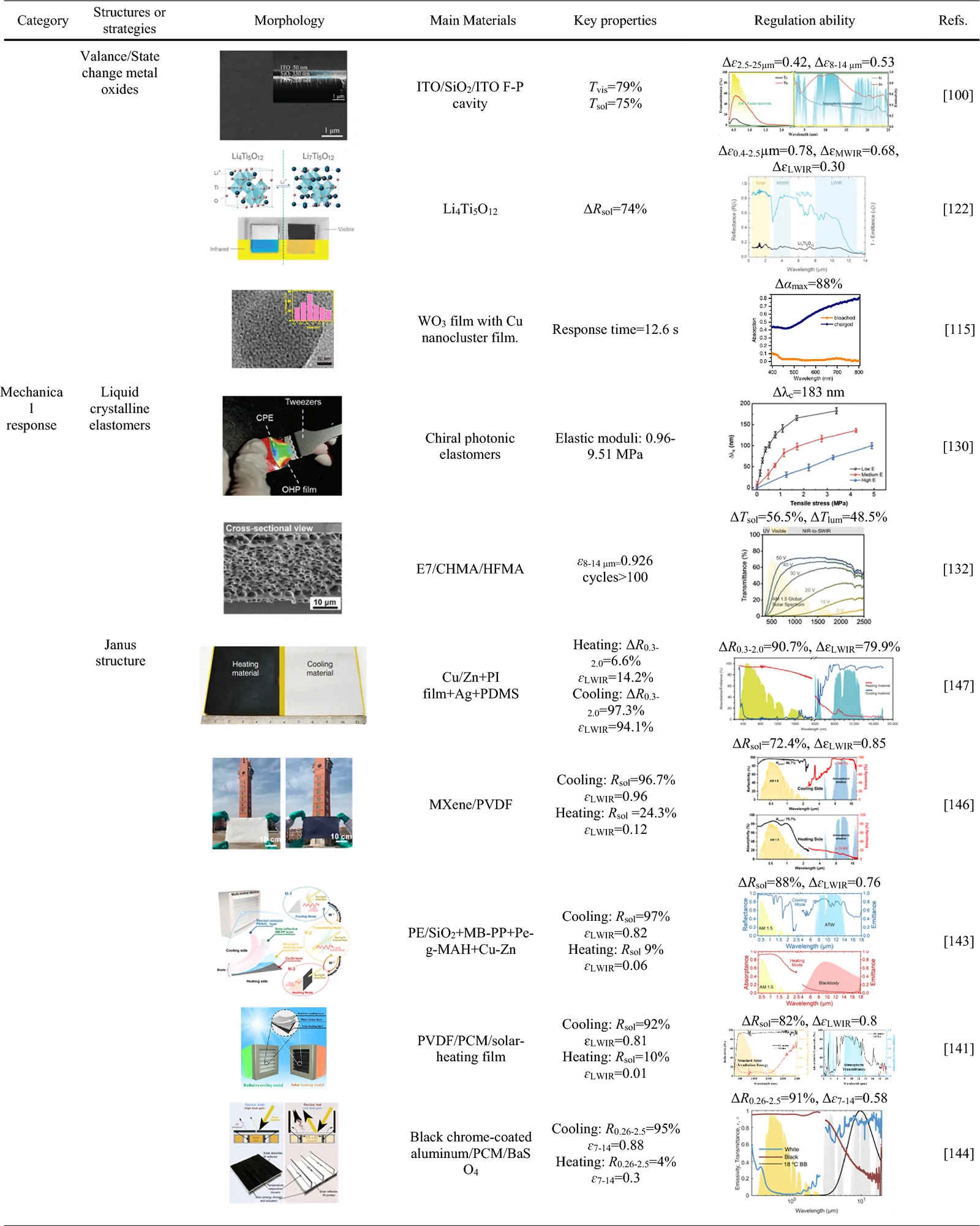

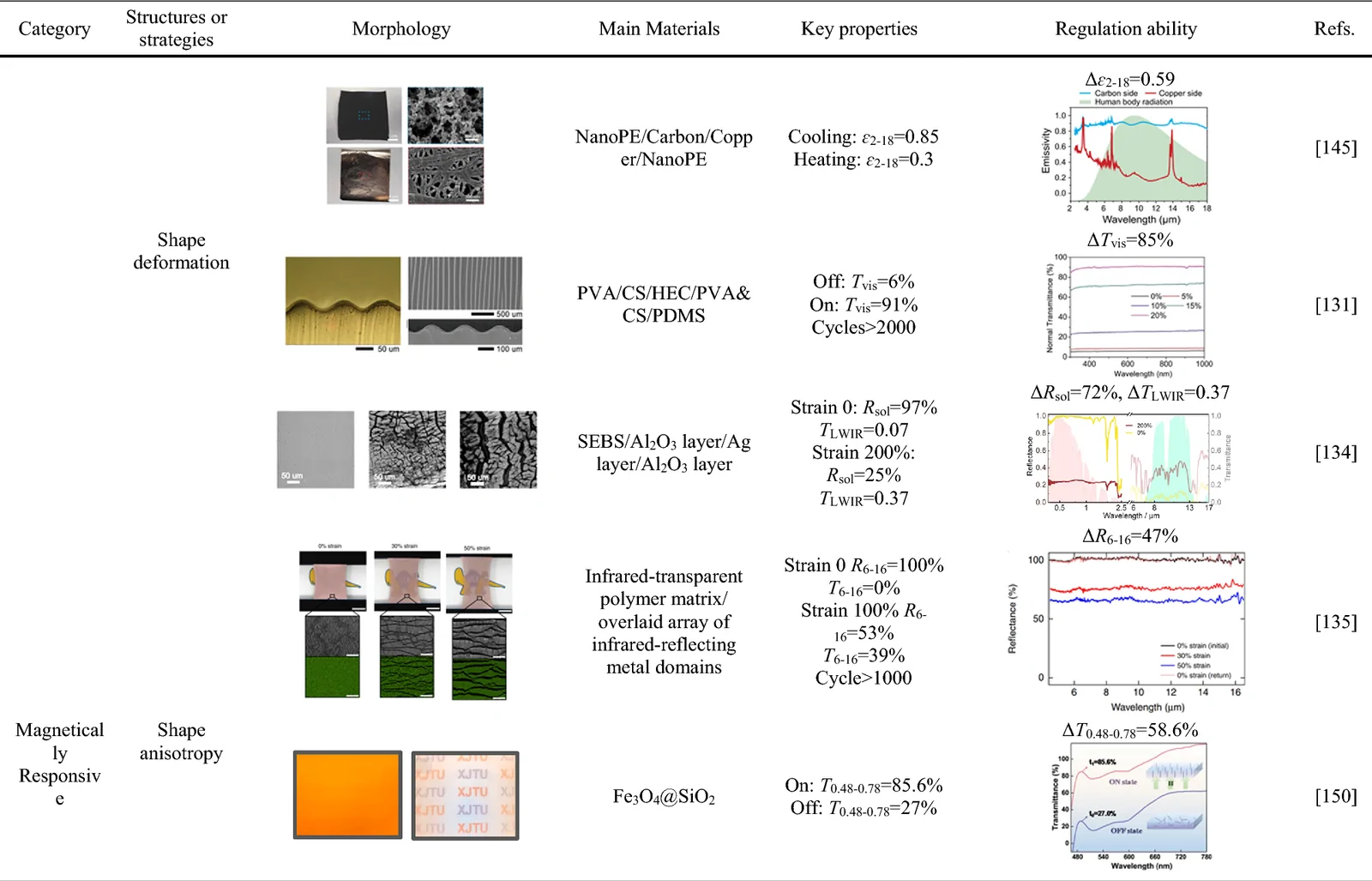
Summary of active response

### Passive Response Structure

Passive response of dynamic radiative thermal management refers to the mechanisms uses the intrinsic material properties or their presentation forms to achieve self-adaptation with changes in ambient temperature, humidity, etc. Passive response does not require additional energy input and has a simple structure and low cost, but the response speed is relatively slow, and the regulation accuracy is limited. For instance, PCM can undergo structural transitions in response to temperature fluctuations, thereby achieving regulation of PRC efficiency. With the growing demand for dynamic control over spectral radiative characteristics, various passive regulation strategies have emerged. Among these, temperature-responsive passive response has been widely promoted due to its direct use of temperature changes to dynamically adjust spectral radiation properties, which can be seamlessly integrated with thermal management systems. This section provides an overview of existing passive DRC technology, with a particular emphasis on thermal response.

#### Thermal Response

Thermally driven response is a dynamic regulation method that uses temperature variations as external stimuli to induce changes in the internal structure, phase state, or chemical properties of materials, thereby achieving dynamic regulation of its optical and thermal radiation characteristics [158]. For instance, elevated temperatures may trigger phase transitions, leading to significant changes in absorption or reflection within specific wavelength regions, or induce thermal expansion effects that modify the material’s microstructure. Thermal-responsive materials can be categorized into two primary mechanisms: One involves direct modifications to the intrinsic material properties, while the other relies on temperature-driven alterations of the dielectric environment. The implementation of the first method mainly relies on thermochromic materials or thermally responsive nanomaterials. Thermochromic materials, such as VO_2_, LC polymers, and thermosensitive dyes, undergo phase transitions or molecular rearrangements within defined temperature ranges, thereby modulating their radiative characteristics. Similarly, LC polymers exhibit changes in molecular ordering at specific temperatures, which directly affect their transparency or scattering behavior. On the other hand, materials with thermal expansion/contraction mechanism (such as thermal-responsive hydrogels and shape memory polymers) undergo volume or morphology changes with temperature, thereby modulating their spectral radiation properties. For example, hydrogels contract upon dehydration at elevated temperatures, leading to microstructural alterations that impact their scattering or absorption properties.

##### VO_2_

**Fig. 8:**
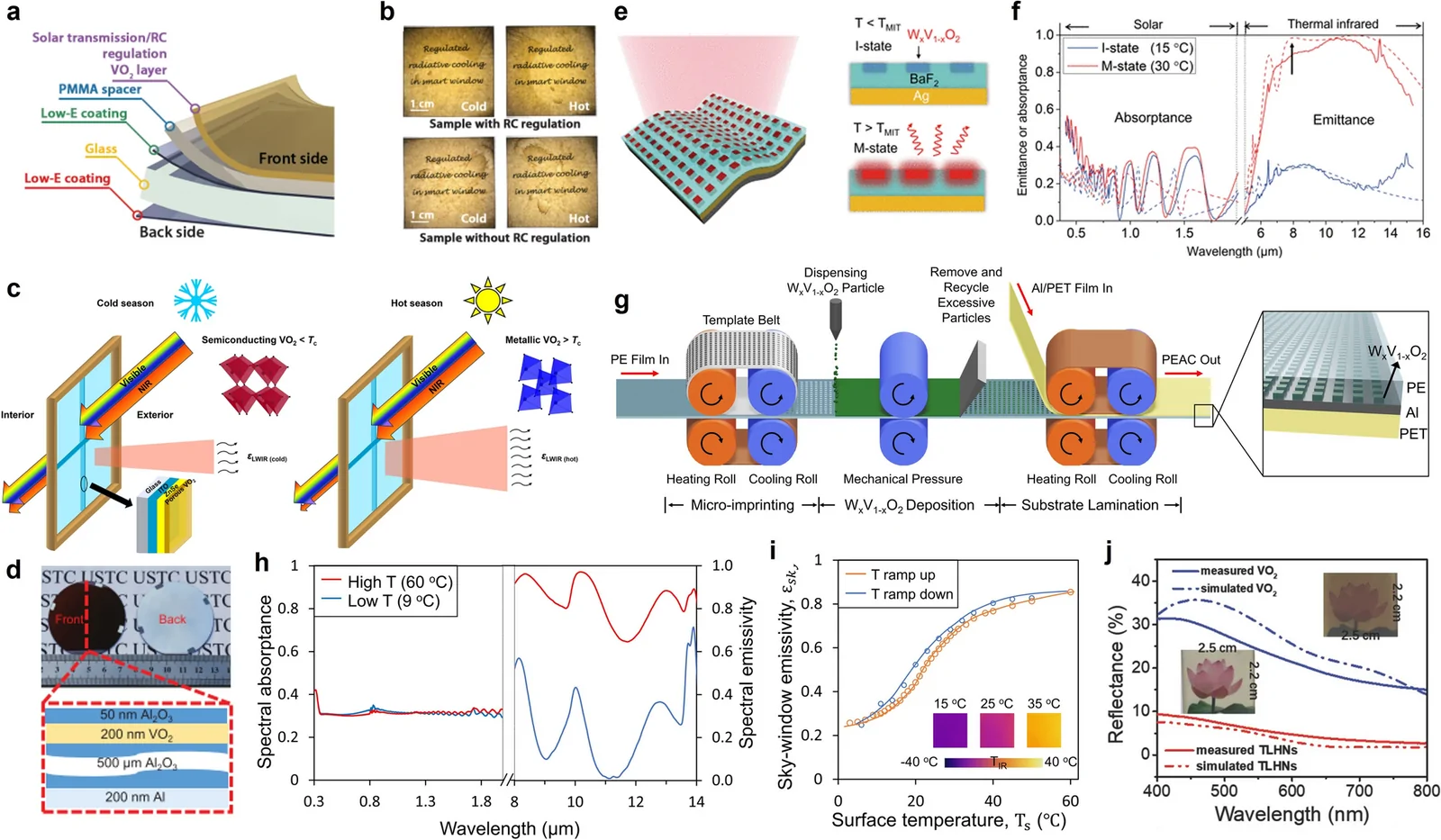
VO_2_-based thermal response DRC materials. **a** Schematic structure and **b** photos of the smart window [13]. Copyright 2021, AAAS. **c** Three-dimensional schematic demonstrates the operation of VO_2_/ZnSe/ITO/Glass RCRT windowpane [162]. Copyright 2024, De Gruyter Brill. **d** Photos and multilayered structure of the spectrally self-adaptive absorber/emitter [163]. Copyright 2022, National Academy of Sciences. **e** Structural schematics and **f** spectral characteristics of the temperature-adaptive radiative coating [12]. Copyright 2021, AAAS. **g** Roll-to-roll printing process, **h** solar absorption and thermal emissivity, and **i** sky-window emissivity of the printable, emissivity-adaptive, and albedo-optimized covering [166]. Copyright 2023, Elsevier. **j** Schematic procedure and reflectance spectra of the SiO_2_/TiO_2_/VO_2_ coatings [170]. Copyright 2018, John Wiley & Sons

VO_2_ is renowned for its reversible temperature-dependent dielectric constants, exhibiting significant disparities between their metallic and insulating states. At a relatively moderate phase transition temperature (68 °C), rutile VO_2_ undergoes a reversible metal–insulator transition to monoclinic VO_2_. These optical properties transition has made VO_2_ an ideal thermochromic material for smart window [159, 160]. VO_2_-based material designs are typically categorized into thin films, metamaterials, and core–shell structures [161]. Long et al. [13] fabricated a PMMA-based thermochromic smart window incorporating tungsten-doped VO_2_ (Fig. 8a). As shown in Fig. 8b, by constructing a Fabry–Perot (F-P) resonator, the smart window achieved a Δ*ɛ*_LWIR_ of 0.4 and an Δ*R*_sol_ of 9.3% under varying temperature conditions. In addition, Long et al. [162] further modulated the F-P resonator’s broadband emissivity by adjusting the porosity of the VO_2_ layer to accommodate diverse climate regions’ energy-saving needs (Fig. 8c). Compared to dense VO_2_, the optimized porous VO_2_ samples exhibit enhanced LWIR emittance contrast (Δ*ε*_Lwɪʀ_ ≥ 0.4) while maintaining a high average visible transmittance (*T*_vis_ = 41%).

For opaque components, thermal regulation requires materials that reflect heat at low temperatures to prevent heat loss and emit heat at high temperatures for cooling. This desired behavior is contrary to the intrinsic phase transition properties of VO_2_ [41]. Pei et al. [163] developed a self-adaptive absorber/emitter for PT and PRC with strong solar absorption and switchable emission within the ATW band (Fig. 8d). Another application of VO_2_ involves creating F-P resonators with an inverse functionality. At high temperatures, a metallic VO_2_ top mirror forms a resonator with high emittance. At low temperatures, VO_2_ becomes transparent, transforming the device into a low-emittance, high-reflectance surface [164]. The phase transition temperature can be finely tuned to ambient levels by varying tungsten (W) doping concentrations [165]. Using this principle, Tang et al. [12] developed a temperature-adaptive DRC radiative cooling using the MIT of W_*x*_V_1−*x*_O_2_. By adjusting the composition (*x* ≈ 1.5%), the transition temperature is tuned to 22 °C. As shown in Fig. 8e, dynamic thermal radiation regulation can be achieved by embedding a patterned W_*x*_V_1−*x*_O_2_ 2D array of F-P resonators in a BaF_2_ dielectric layer on an Ag film. Figure 8f illustrates that the temperature-adaptive DRC achieves a solar absorptivity of 25% while the *ε*_LWIR_ increases from 0.20 in the insulating state to 0.90 in the metallic state. Similarly, Li et al. [166] designed a covering structure composed of a PE layer, a periodic array of W_*x*_V_1−*x*_O_2_ blocks, an Al bottom layer, and a PET substrate (Fig. 8g). This covering, fabricated using roll-to-roll manufacturing and recyclable materials, exhibits significant emittance variation between heating and cooling modes while maintaining nearly constant solar absorptance. As shown in Fig. 8h, i, the PEAC system can switch *ɛ*_LWIR_ from 0.25 to 0.85.

VO_2_-based core–shell nanostructures can be categorized into bilayer and trilayer architectures, where VO_2_ is encapsulated within a dielectric shell (e.g., SiO_2_, TiO_2_, and Al_2_O_3_) or a metallic shell (e.g., Au and Ag) [167]. Xie et al. [168] investigated the impact of the shell’s optical constants and thickness on VO_2_’s light transmittance and solar regulation using effective medium theory combined with the transfer matrix method. Wu et al. [169] proposed an thermal control coating based on CaF_2_/VO_2_ core–shell microspheres. This coating exhibits a reversible increase in emittance from 0.47 at 30 °C to 0.83 at 90 °C. Yao et al. [170] synthesized SiO_2_/TiO_2_/VO_2_ trilayer hollow nanospheres and developed multifunctional coatings based on this structure. As shown in Fig. 8j, the coating displays exceptional optical performance with a *T*_vis_ of 74% and a Δ*T*_sol_ of 12%.

##### Thermochromic Materials

PRC technology is typically achieved using white materials with high *R*_solar_ to maximize cooling efficiency. However, their broadband reflectance in the visible spectrum limits practical applications. For instance, white coatings may be esthetically or functionally unsuitable for buildings or other structures, and their high reflectance poses challenges in adapting to dynamic environments [171]. To address these limitations, researchers have developed colored PRC materials [172]. Thermochromic microcapsules, a key component of colored PRC materials, consist of a shell material and a core comprising organic dyes, color developers, and solvents. At lower temperatures, the thermochromic dye accepts electrons from the color developer, displaying a specific color. As the temperature rises, the solvent mixture gradually melts, dissolving the color developer and separating it from the dye [173, 174]. As a result, the color of the microcapsule fades. In colored PRC materials, the VIS spectrum is selectively absorbed to present the desired color, while other wavelengths are reflected [175, 176].

**Fig. 9:**
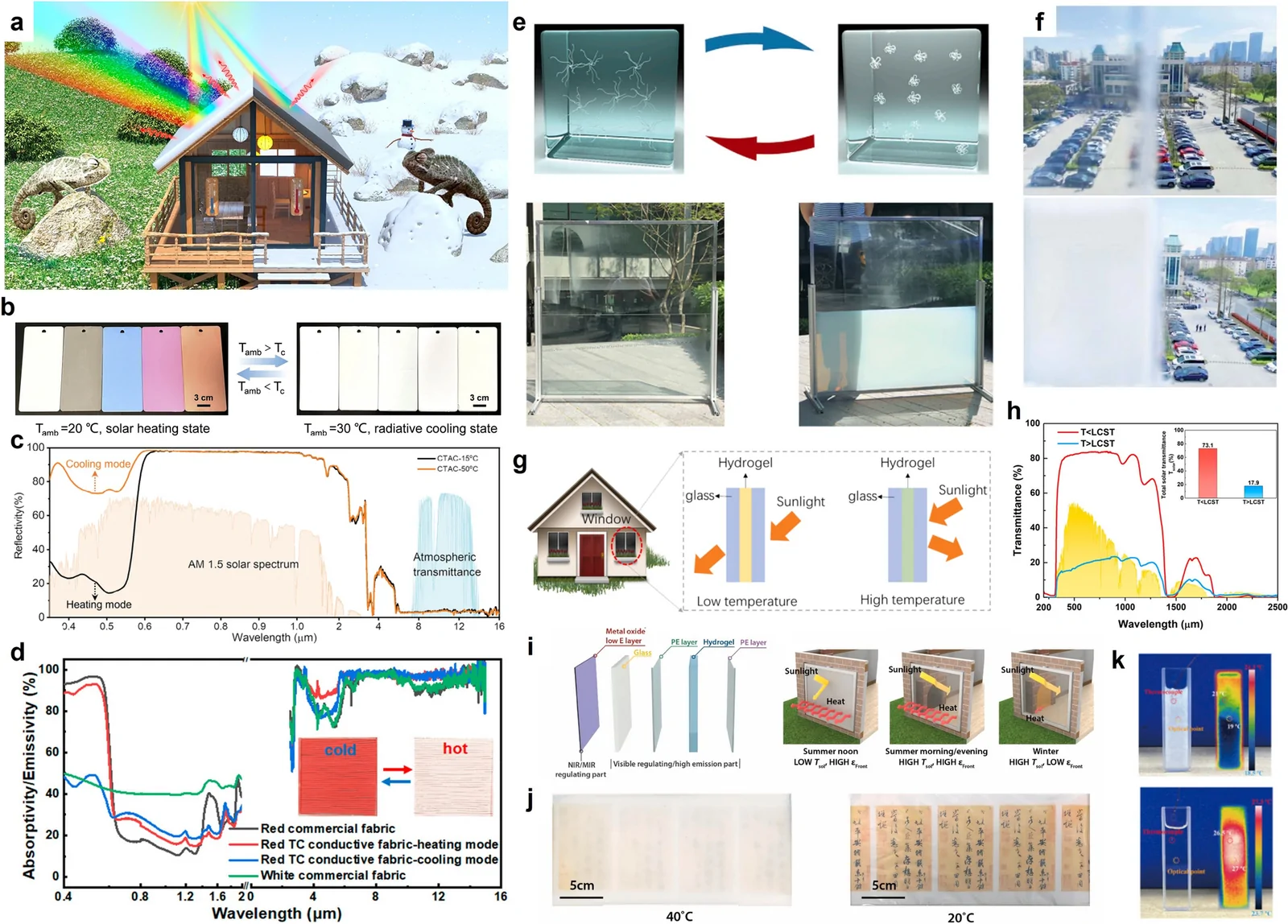
Thermal response DRC materials based on thermochromic materials. **a** Temperature-adaptive radiative cooling coating inspired by Namibian chameleon [177]. Copyright 2023, ACS Publications. **b** PRC coating with thermochromic materials at different temperatures [178]. Copyright 2022, Elsevier. **c** Measured reflectivity of dual-mode colored temperature-adaptive cloak [179]. Copyright 2024, John Wiley & Sons. **d** Spectral characteristics and morphology of the thermochromic conductive fiber [181]. Copyright 2023, ACS Publications. **e** Structure and optical photos of smart window at different temperatures [182]. Copyright 2020, Elsevier. **f** Optical photos of the PVDF@PNIPAm film at 20 °C and 40 °C [183]. Copyright 2020, Royal Society of Chemistry. **g** The structure and sunlight transportation of the proposed thermal-responsive smart window [184]. Copyright 2025, Elsevier. **h** Comparison of solar transmittance before and after phase transition of pNIPAm hydrogel [185]. Copyright 2021, ACS Publications. **i** Working principle and **j** optical photo of tunable emissivity thermochromic smart window at different temperatures [186]. Copyright 2021, Elsevier. **k** Transmittance and temperature of paraffin at different temperatures and phases [187]. Copyright 2022, Elsevier

Dong et al. [177], inspired by the temperature adaptability of Namibian chameleons, combined biomimetic design with radiative thermal regulation and pioneered a novel approach integrating temperature-adaptive solar absorption with PRC technology (Fig. 9a). The temperature-adaptive PRC coating they designed and fabricated achieves a visible light modulation capacity of 41% while maintaining a 93% emittance within the LWIR. Wang et al. [178] thermochromic materials into PRC coatings, producing adaptive and colorful solar heating and PRC coatings (as shown in Fig. 9b). This method allows for tunable phase change temperatures and a wide range of colors, enhancing the versatility of the coating. Yin et al. [179] proposed a colored temperature-adaptive cloak. As illustrated in Fig. 9c, the cloak consists of a color functional top layer constructed of thermochromic microcapsules and fluorescent dyes and a PRC bottom layer. The color top layer is responsible for color display by reflecting light in the desired color in the visible light wavelength range and has controllable solar reflection in response to temperature fluctuations. Similarly, Son et al. [180] developed a dual-layer material where the bottom layer comprises PVDF and Al_2_O_3_ particles to reflect maximum sunlight, while the top layer contains thermochromic pigments that display different colors depending on temperature. Ma et al. [181] developed a thermochromic conductive fiber with a coaxial structure consisting of a conductive core and a thermochromic outer shell. As shown in Fig. 9d, compared to commercially available colored textiles, the TC fiber-based fabric exhibits consistent color in cold environments but transitions to a white appearance in hot conditions, facilitating adaptive thermal management.

##### Hydrogels

Hydrogels represent another extensively studied class of thermal response materials for regulating spectral characteristics [188, 189]. Hydrogel polymers are dispersed in water molecules below the critical temperature and generate high solar transmittance. Conversely, when the temperature exceeds the critical temperature, the hydrogen bonds within the hydrogel break, causing the polymers to become hydrophobic. This transition induces polymer aggregation and the formation of polymer clusters, leading to phase separation and strong internal scattering, which significantly reduces solar transmittance. Recent studies have integrated hydrogels with PRC materials that exhibit consistently high *ε*_LWIR_, such as PVDF, PDMS, and PMMA. This integration has expanded the functional capabilities of hydrogel-based smart windows, enhancing their potential for dynamic radiative thermal management.

Long et al. [182] developed a thermally responsive smart window by trapping thermochromic the poly(N-isopropylacrylamide) (PNIPAm) hydrogel-derived liquid within glass. As illustrated in Fig. 9e, the material exhibits remarkable thermally responsive optical properties, including 90% light transmittance and 68.1% solar modulation. This results in a significant transparency difference around the critical solution temperature, making it ideal for dynamic radiative thermal management. Wu et al. [183] designed a sandwich-structured adaptive film composed of PNIPAm hydrogel and a PVDF film. This film demonstrates significant visible light reflection/transmission modulation (Δ*R*_vis_ = 70.0% and Δ*T*_vis_ = 86.3%) and a high *ε*_LWIR_ (0.96). As shown in Fig. 9f, outdoor tests reveal dramatic changes in optical transparency with temperature fluctuations. At low temperatures, the view through the film remains clear, while at high temperatures, the view becomes obscured, offering privacy and thermal regulation. Zhao et al. [184] proposed a temperature-responsive smart window by introducing hydrogels that modulate optical properties in response to thermal stimuli. As shown in Fig. 9g, this window exhibits a switchable solar transmittance that varies from 0% (hot state) to 78% (cool state) across the solar spectrum, enabling dynamic solar energy regulation. Fang et al. [185] introduced a pNIPAm/PET/Cr sandwich-structured thermal homeostasis. As depicted in Fig. 9h, the total solar transmittance of the pNIPAm hydrogel decreases from 73.1% to 17.9% across the critical solution temperature, and with a Δ*T*_solar_ of 55.2%. Long et al. [186] developed a tunable emittance thermochromic smart window with a Low-E/glass/PE/HPC/PE structure that can simultaneously regulating solar transmission and thermal radiation. As shown in Fig. 9i, j, this smart window demonstrates a Δ*T*_lum_ of 71.6% and 50.3% Δ*T*_sol_ at room temperature. By reversing the window panel, the window achieves high *ε*_LWIR_ (0.95) in summer for efficient heat dissipation and low *ε*_LWIR_ (0.1) in winter to retain indoor heat.

Similar to the function of hydrogel, another approach to modulate radiative properties in the visible wavelength range involves using solid–liquid PCM [190, 191], whose optical characteristics exhibit significant changes near their phase change temperature [192, 193]. This property enables efficient thermal regulation across different temperature conditions. Su et al. [187] investigated the temperature-dependent radiative properties of paraffin wax, a commonly used PCM. As illustrated in Fig. 9k, paraffin exhibits distinct emittance changes between its solid and liquid states, resulting in a Δ*T* of 85% in the 0.19–1.1 μm range and 41.1% in the 8–13 μm range.

#### Photodriven Response

The primary factor influencing thermally driven regulation is the ambient temperature. Beyond temperature responsiveness, light intensity can also serve as a stimulus for modulation [194]. Photodriven response is a dynamic approach that utilizes light to trigger structural or chemical state changes in materials. Photochromic materials undergo molecular isomerization or chemical transformations under illumination at specific wavelengths, leading to alterations in their optical characteristics. Additionally, photothermal nanomaterials, such as gold nanorods and carbon nanotubes, generate localized thermal effects upon exposure to light, which can modify their surface plasmon resonance properties or induce thermal expansion, enabling dynamic tuning of optical behavior [195].

**Fig. 10:**
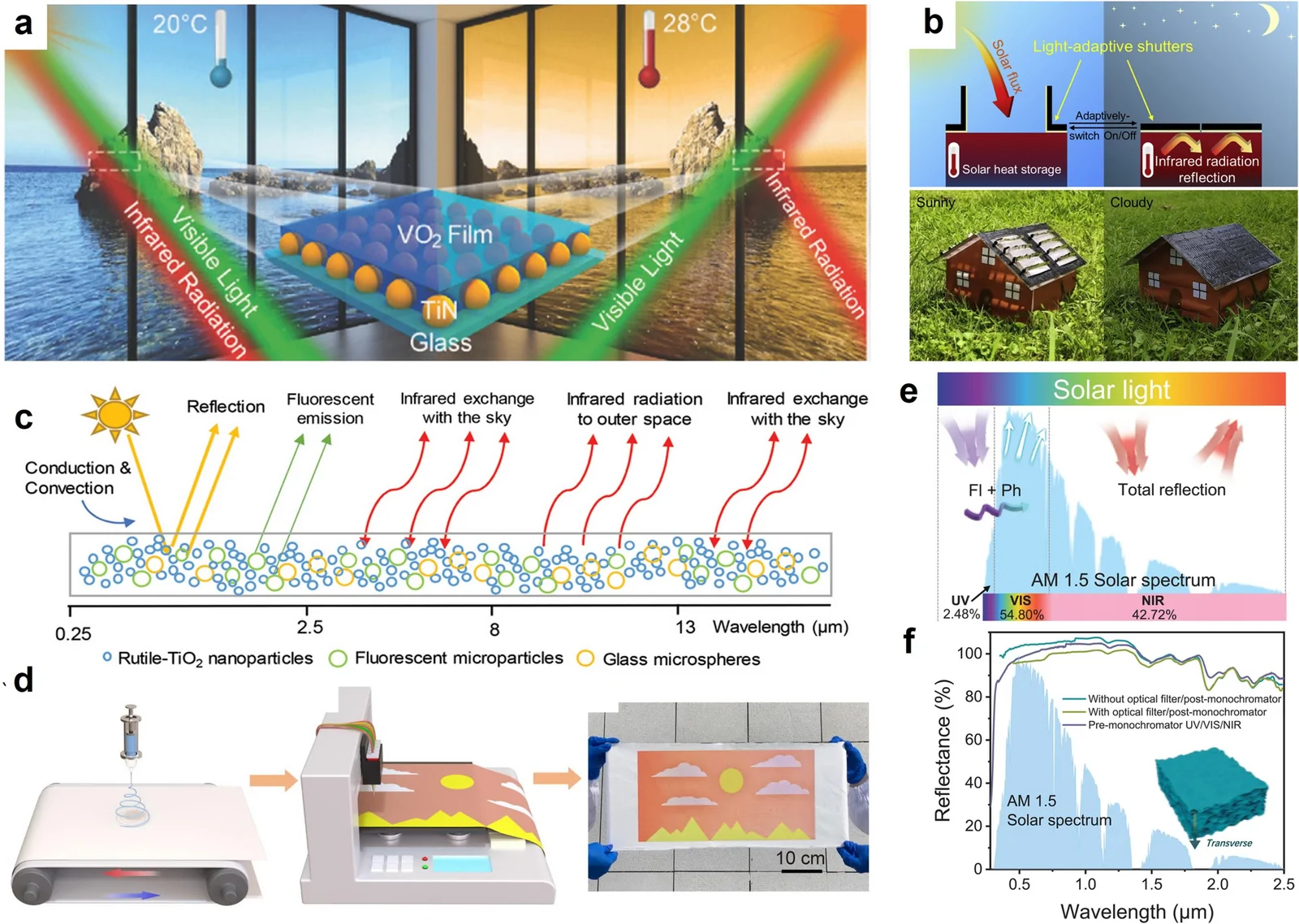
Photodriven response DRC materials. **a** Schematic representation of hybrid VO_2_/TiN material applied as an intelligent window coating [196]. Copyright 2018, John Wiley & Sons. **b** Schematics mechanism and photos of the bioinspired light-adaptive shutter [197]. Copyright 2021, Elsevier. **c** Schematics of the cooling mechanism of the self-adaptive DRC coating [200]. Copyright 2020, John Wiley & Sons. **d** Schematic of the electrostatic-spinning setup for producing CA nanofibers film [201]. Copyright 2022, Elsevier. **e** Diagram and **f** reflectance spectra of the photoluminescent hydrogen-bonded biomass aerogel [202]. Copyright 2024, AAAS

Hao et al. [196] developed a smart coating by hybridizing thermochromic VO_2_ with plasmonic TiN nanoparticles. It exhibits infrared regulation properties, as shown in Fig. 10a, blocking infrared radiation under strong illumination at 28 °C, while remaining infrared transparent under weak irradiation or at a low temperature of 20 °C. The VO_2_/TiN coatings achieve an integral *T*_vis_ of 51% and demonstrate excellent infrared switching efficiency of 48% at 2000 nm, making them promising for dynamic radiative thermal management. Zhou et al. [197] introduced a bioinspired light-adaptive shutter with a multilayer structure that autonomously toggles between open and closed states due to the photothermal expansion mismatch effect. As depicted in Fig. 10b, this shutter, when integrated into a solar thermal storage system, governs the incident and dissipated radiation, and achieving near-zero net radiative heat loss. As a special case of photodriven regulation, fluorescent materials with upconversion or downconversion capabilities have also been employed for the regulation of radiative properties [198, 199]. Fan et al. [200] achieved PRC by integrating particle scattering, solar-excited fluorescence, and mid-infrared broadband radiation. As illustrated in Fig. 10c, this advanced coating provides daytime cooling and mitigates nighttime overcooling. Under direct sunlight with a solar intensity of 850 W m^−2^, the coated aluminum panel maintains a surface temperature 6 °C below the ambient temperature, effectively improving thermal management in architectural applications. Zhu et al. [201] demonstrated a photoluminescent-based colored PRC with high internal quantum efficiency, capable of achieving sub-ambient cooling across the full color spectrum. As shown in Fig. 10d, they developed a scalable electrostatic-spinning/inkjet printing method to fabricate the film. The quantum dot layer converts UV–VIS light into emission wavelengths, thereby minimizing solar heat gain, while the CA nanofiber substrate reflects sunlight and facilitates thermal dissipation. Building on this work, Zhu et al. [195] proposed a photosynthetically active PRC film that lowers ambient air temperature, reduces water evaporation, and enhances photosynthesis in dryland plants. This film consists of a photonic crystal layer sandwiched between PDMS layer and PAM antifogging layer. The photonic crystal selectively transmits photosynthetically active sunlight with 71% transmittance at 0.4–0.5 μm and 77% at 0.6–0.7 μm, optimizing conditions for plant growth while maintaining effective radiative cooling. Zhao et al. [202] showed an intrinsic photoluminescent biomass aerogel, which uses the phosphorescence and fluorescence characteristics generated by the synergistic interaction between gelatin and DNA to convert UV light to VIS light (Fig. 10e). By using a postmonochromator UV–VIS–NIR device, the received light from photoluminescence is identified and detected. Due to the energy conversion, the aerogel receives 104.0% of the energy in the VIS spectrum (400–800 nm) (Fig. 10f).

#### Spatial Structure Regulation

##### Pore Structure

**Fig. 11:**
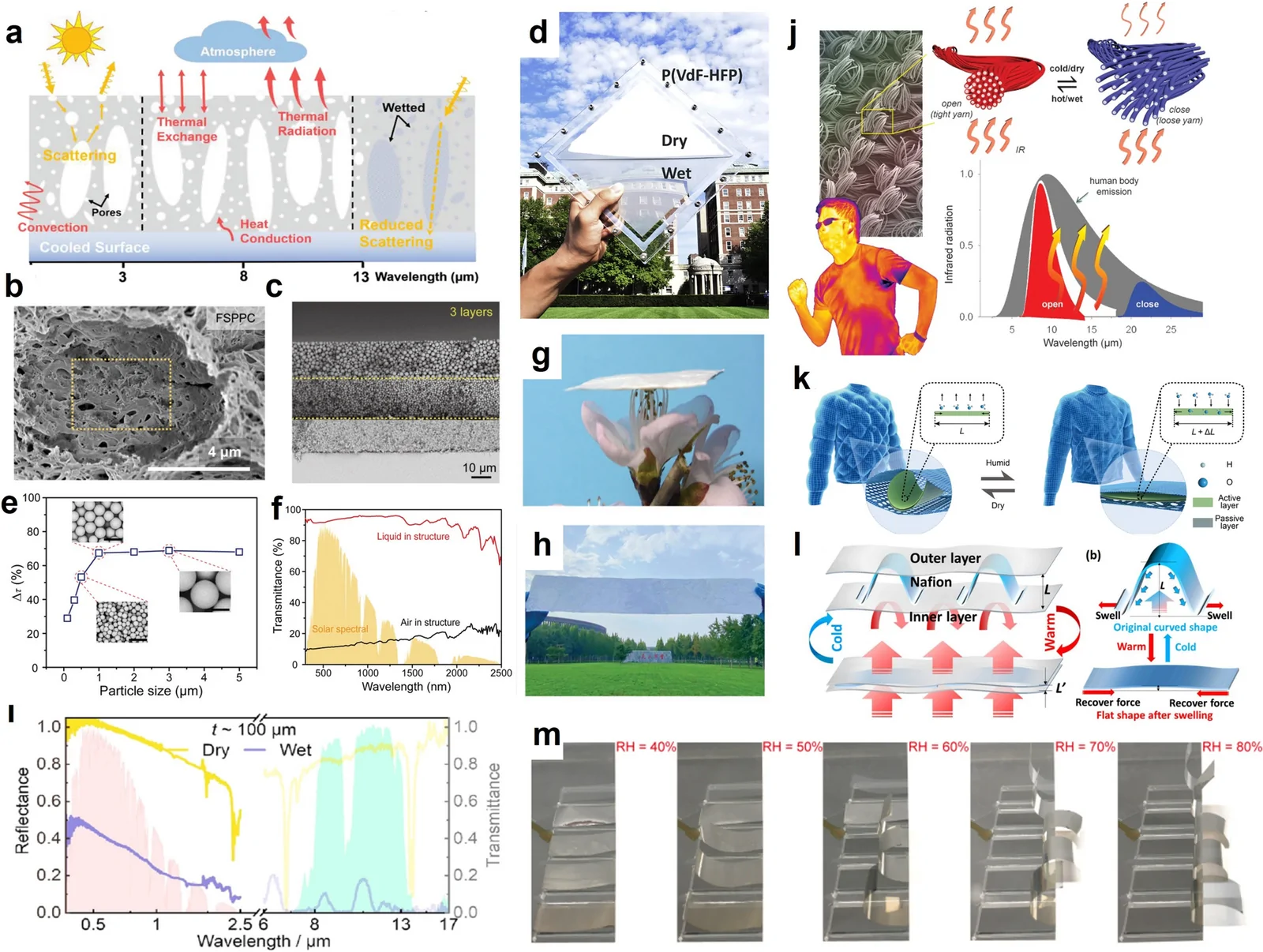
DRC materials based on spatial structure. **a** Schematic illustration of working principle of designed hierarchical porous structure [210]. Copyright 2022, John Wiley & Sons. **b**, **c** Morphology of the polymer film [208, 209]. Copyright 2024, Springer Nature. Copyright 2022, John Wiley & Sons. **d** Photograph of the system showing dry and wet states [211]. Copyright 2019, Elsevier. **e**, **f** Transmittance performance of the DRC device at different working states [209]. Copyright 2022, John Wiley & Sons. **g**, **h** Photographs of Bio-RC films [212]. Copyright 2023, John Wiley & Sons. **i** Spectral reflectance and transmittance of PE film in dried and wetted states [213]. Copyright 2024, American Chemical Society. **j** Design principles of an infrared gating textile [223]. Copyright 2019, AAAS. **k** Working rationale of the adaptive clothing [218]. Copyright 2025, AAAS. **l** Schematic of bendable smart clothing [220]. Copyright 2017, Springer Nature. **m** Photos of the bending process of nylon-Ag actuators over different humidities [221]. Copyright 2021, AAAS

As described in Sect. 3.1, the introduction of a dielectric function difference interface with the same scale as the incident wavelength in the electromagnetic wave propagation path can cause the electromagnetic wave propagation path to deflect. One of the simplest methods to achieve spatially dynamic radiative regulation is to immerse porous materials, with a refractive index similar to the bulk material, into a liquid medium [203, 204]. As illustrated in Fig. 11a, the high refractive index contrast between the material and air at the pore boundaries induces strong Mie scattering effects when the pore size is comparable to the incident wavelength, which leads to high solar reflectance [205]. However, when the pores are filled with a liquid that matches the material’s refractive index, the sharp reduction in refractive index contrast weakens the Mie scattering efficiency [206]. Dynamic radiative thermal management can be achieved by combining this mechanism with humidity-responsive porous materials [207].

As shown in Fig. 11b and c, the porous structures generally divided into two categories: particle-based and polymer-based structures [208, 209]. Fie et al. [210] reported a single-layer coating with a multi-level porous structure capable of rapid switching between high *R*_sol_ (96.6%) and high *T*_sol_ (86.6%). In its dry state, the coating exhibits high *ɛ*_LWIR_ (0.96), enabling PRC even under harsh tropical climates. Upon wetting, the coating becomes highly transparent across the solar band, facilitating solar heating and providing switchable thermal regulation. Mandal et al. [211] introduced a porous polymer coating, as illustrated in Fig. 11d, which exhibits reversible optical transmittance changes upon wetting with ordinary liquids. At solar wavelengths, reduced light scattering during wetting shifts the coating from a reflective to a transparent state. Deng et al. [209] developed a dynamic radiative thermal management device by utilizing a porous SiO_2_ coating combined with a refractive index-matching liquid to regulate solar transmittance and reflectance. The graded structure, created through heterogeneous particle sizes, enhances scattering efficiency, enabling 80% modulation of solar transmittance (Fig. 11e, f). Feng et al. [212] designed a flexible PRC material with switchable solar transmittance by entangling silica microspheres of varying sizes with bacterial cellulose. This lightweight and scalable material shows promising applications (Fig. 11g, h). Chen et al. [213] engineered a PE film with dynamic solar and thermal regulation. The 100 µm-thick PE film demonstrates outstanding solar modulation, varying from 92% (dry state) to 32% (wet state), and thermal regulation, shifting from 0.86 (dry state) to 0.05 (wet state) (Fig. 11i).

##### Shape Deformation

In addition to the temperature- or humidity-dependent reversible pore structures described above, shape memory materials offer an alternative approach to macro-scale reversible thermal radiation regulation [214]. This strategy is particularly beneficial for personal thermal management [215]. A simple implementation involves thermal switches, such as nickel–titanium alloy springs, which alternate between a low thermal resistance (open) and high thermal resistance (closed) state [216]. For temperature-induced deformation, materials like polypropylene use their thermal expansion properties to regulate thermal radiation. Additionally, dynamic transmittance switching can be achieved by exploiting the photon bandgap variations in expanded polymer clusters. Shape memory structures can also be combined with humidity control mechanisms to prevent radiative heat loss and improve heating efficiency. Under humid conditions, flaps automatically open to promote convection, radiative, and sweat evaporation, thus facilitating cooling. The inclusion of a metal layer not only enhances the flexibility of materials like nylon but also suppresses mid-infrared emission from the human body [217].

Fan et al. [216] proposed a switchable DRC structure consisting of a PRC coating and a temperature-responsive component. In hot weather, this structure transitions to a low thermal resistance state, enabling internal heat dissipation. In cold weather, it shifts to a high thermal resistance state, effectively inhibiting heat loss. Li et al. [218, 219] proposed an adaptive warm cloth, featuring a filling made of a natural bacterial cellulose membrane that responds to human sweating. As shown in Fig. 11k, the thickness of the fabric can be automatically adjusted from 13 mm (low humidity) to 2 mm (high humidity), and its thermal regulation ability has been improved by 82.8%. Similarly, Zhong et al. [220] designed a Nafion-based smart clothing structures that rapidly and reversibly alter their pore size and insulation properties in response to humidity changes (Fig. 11l). Li et al. [221] demonstrated a multimodal adaptive wearable with moisture-responsive flaps composed of a nylon/metal heterostructure. As shown in Fig. 11m, this design simultaneously regulates convection, sweat evaporation, and MIR emission, enabling rapid and large-scale heat transfer in response to human perspiration vapor, and expanding the thermal comfort zone by 30.7% compared to traditional static textiles. Zhao et al. [222] reported a bimorph textile actuator consists of polypropylene and MXene. Due to the opposing thermal expansion of the two layers and the enhanced photothermal efficiency of MXene, the actuator exhibits effective deformation (1.38 cm^−1^) under low solar power conditions (100 mW cm^−2^). Zhang et al. [223] constructed an infrared-adaptive textile composed of carbon nanotube. As shown in Fig. 11j, these fibers expand and collapse under temperature and humidity stimuli, altering the internal pore distribution and enabling 35% modulation of infrared emittance, facilitating dynamic thermal regulation in wearable applications. Similarly, Hu et al. [224] developed a dual-layer fabric designed to simultaneously manage sweat and cooling. This fabric consists of hydrophobic PET on the one side and hydrophilic cellulose fibers on the other, achieving high infrared transmittance while maintaining thermal-moisture comfort. The detailed information on various passive response structure are summarized in Table 2.

**Table 2:**
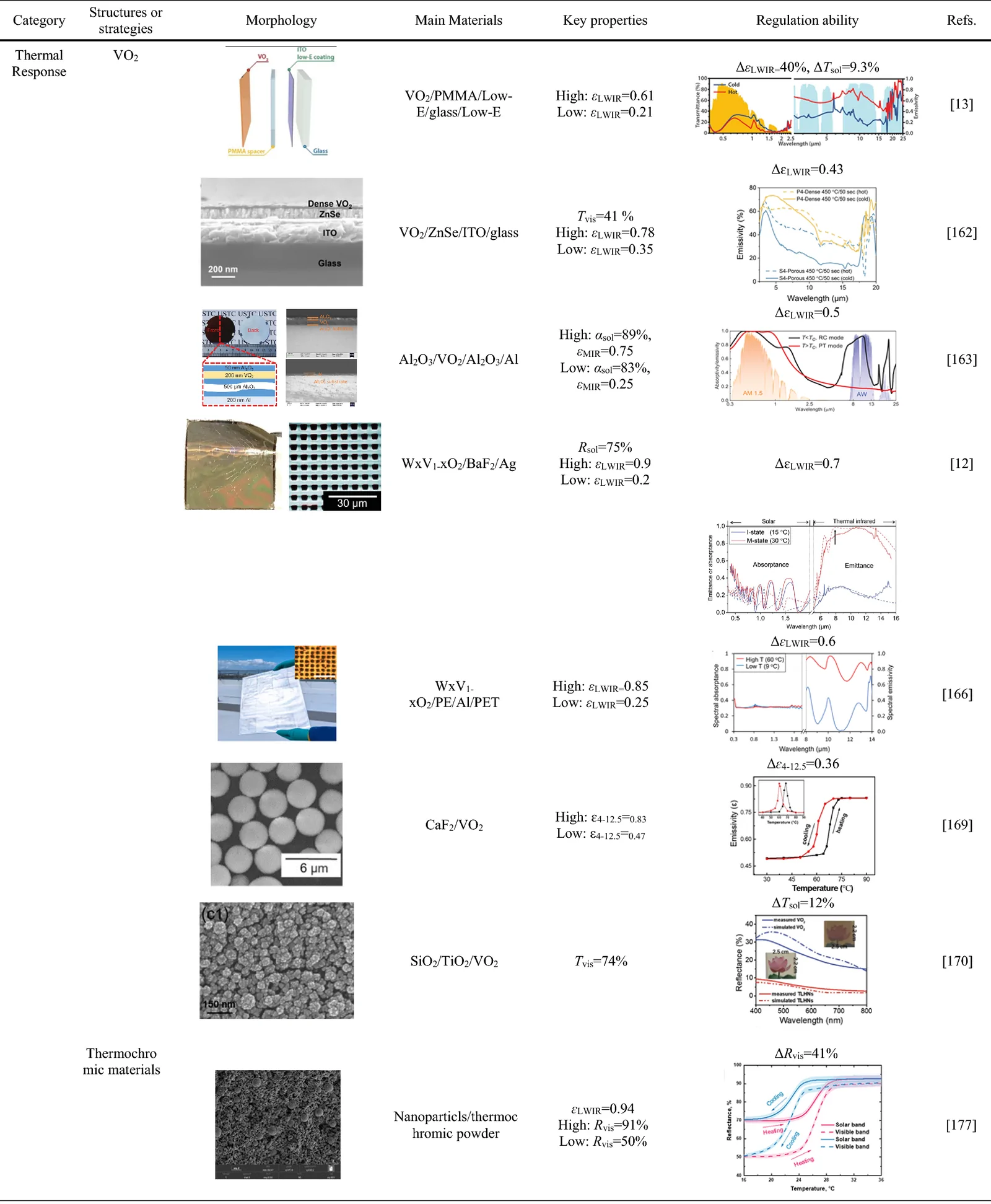

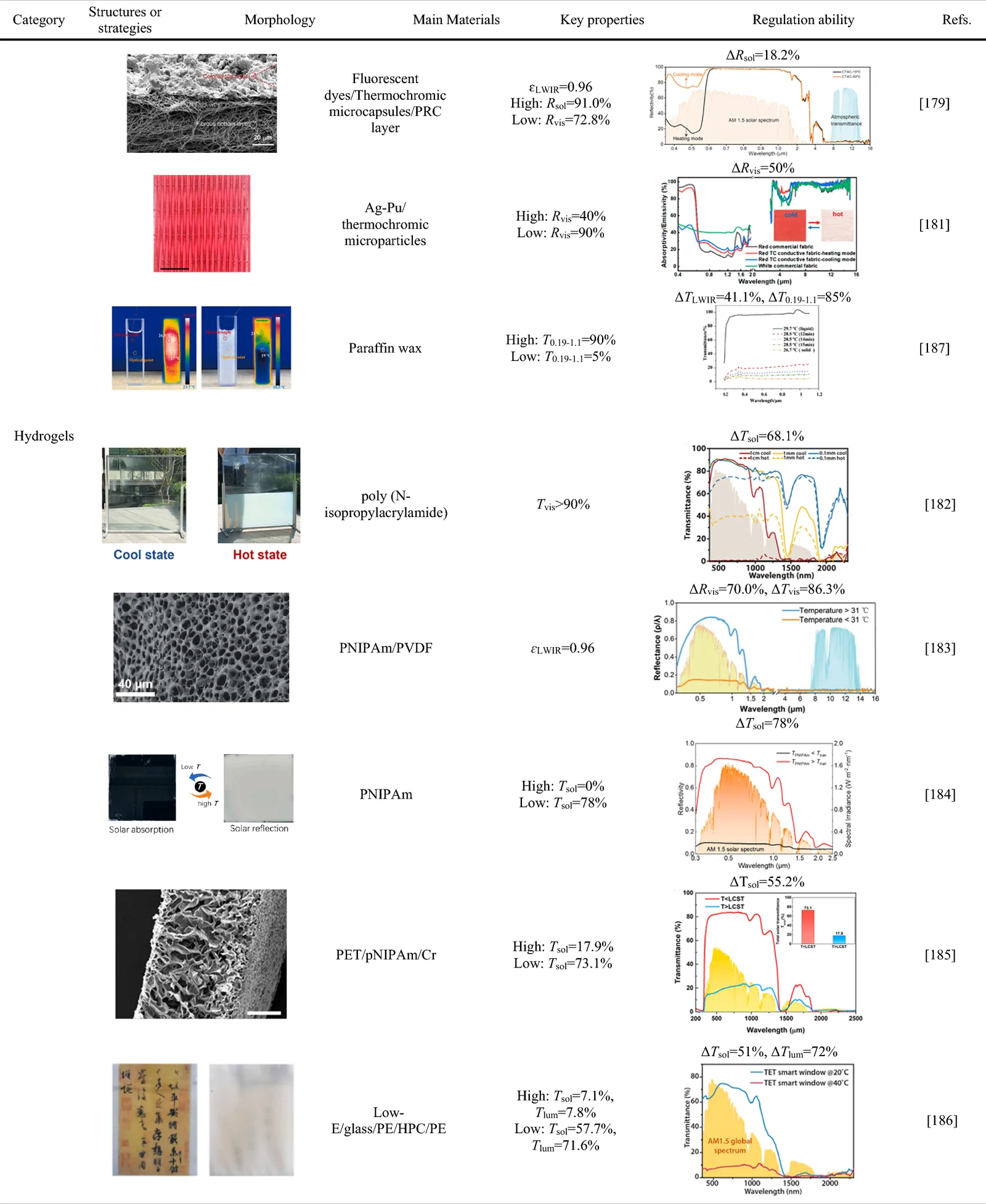

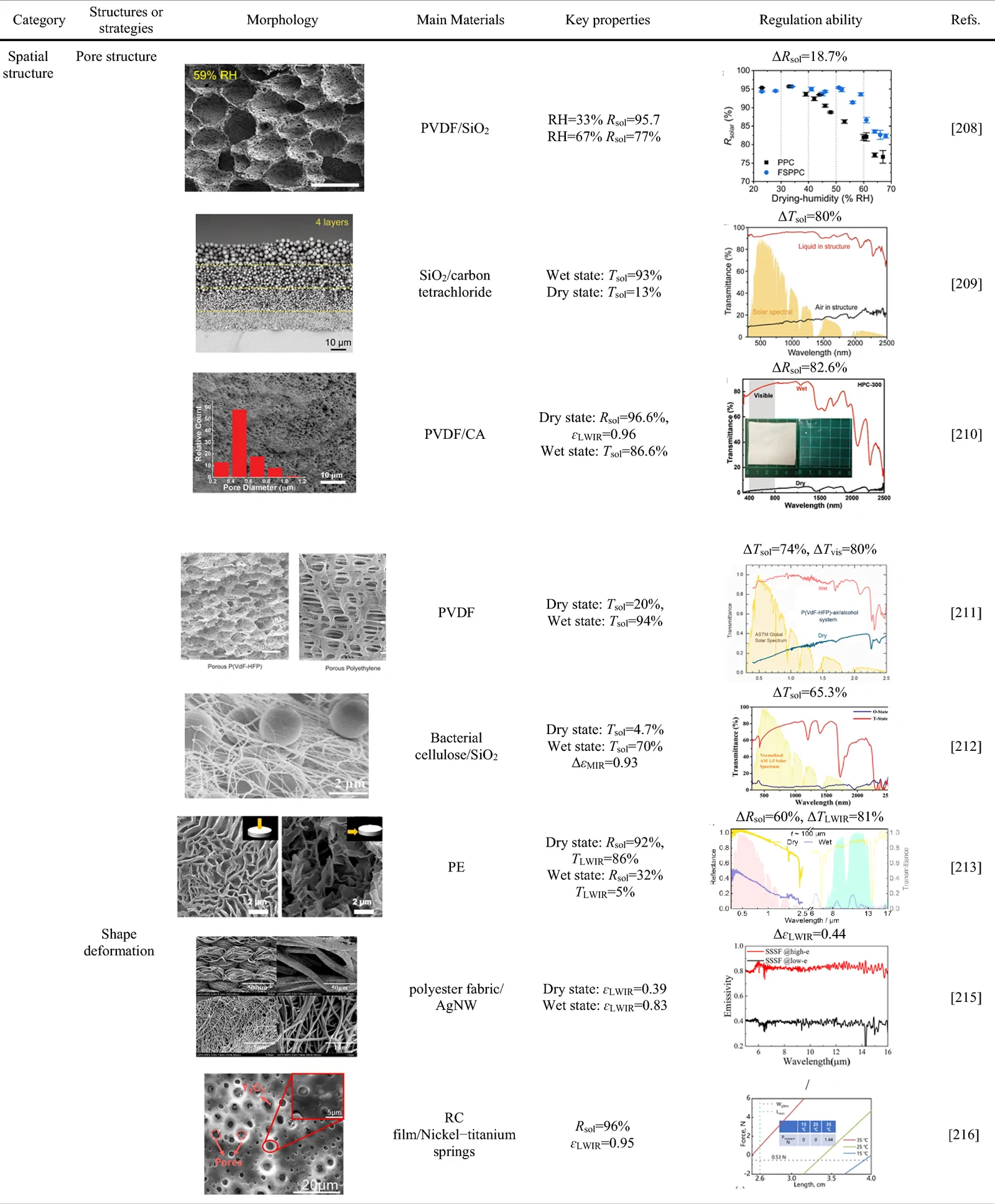
Summary of passive response

## Multi-stimuli Response

### Significance of Multi-band Radiation Regulation

Over the past decade, radiative thermal regulation has witnessed remarkable advancements. However, the application of single-mode regulation in complex environments remains constrained by various limitations. For instance, while electrochromic material can rapidly modulate solar spectrum, it exerts minimal influence over the infrared emittance. Humidity-responsive materials, though highly sensitive to environmental changes, suffer from relatively slow response times. Consequently, the synergistic integration of multiple regulation mechanisms (such as electrical, optical, thermal, humidity-driven, and mechanical stimuli) has emerged as a pivotal strategy for achieving broadband, adaptive thermal management. The core of this technology is to construct a composite structure that can respond to various external stimuli, which not only enhances performance optimization but also significantly broadens the scope of practical applications [225].

The principal advantage of multi-band dynamic radiative thermal management is its ability to adapt to complex environmental conditions. In the field of building energy efficiency, an ideal smart window should reflect solar radiation while enhancing infrared emission for passive cooling in summer, whereas in winter, it should facilitate solar absorption while minimizing thermal losses. A single regulatory mechanism struggles to fulfill these competing demands simultaneously. However, by integrating electrical and thermal control with optical coatings, selective spectral regulation can be effectively achieved. Similarly, military camouflage systems operating under multi-spectral reconnaissance require dynamic regulation spanning from the visible to mid- and far-infrared wavelengths. By combining different regulatory strategies, materials and devices can transcend the constraints of individual mechanisms, offering tunable thermal radiation properties over an extended spectral range. Furthermore, the integration of multiple regulation modes enhances both dynamic response capability and energy efficiency. Electrically driven approaches require continuous energy input for rapid actuation. Humidity- or temperature-responsive materials are energy efficient but have slow response times. The hybridization of these methods offers a complementary advantage: electrical or optical activation can be employed for rapid adjustments, while passive stimuli-driven maintain stability in steady state conditions, thereby reducing overall energy consumption. Likewise, bioinspired material designs have further advanced the development of DRC technology [226], as seen in chameleon-inspired multilayer structures, which use the interplay between chemical tuning and mechanical deformation to achieve rapid and large-scale optical property regulation.

### Multi-stimuli Response Structure

Among the various dynamic regulation strategies, thermal-responsive materials have garnered significant attention due to its ability to directly utilize temperature variations for dynamic radiative control and seamlessly integrate with thermal management systems. Existing approaches that couple multiple regulation mechanisms primarily build upon temperature-responsive radiative control while incorporating additional stimuli such as mechanical flipping, pressure, and electrical excitation.

**Fig. 12:**
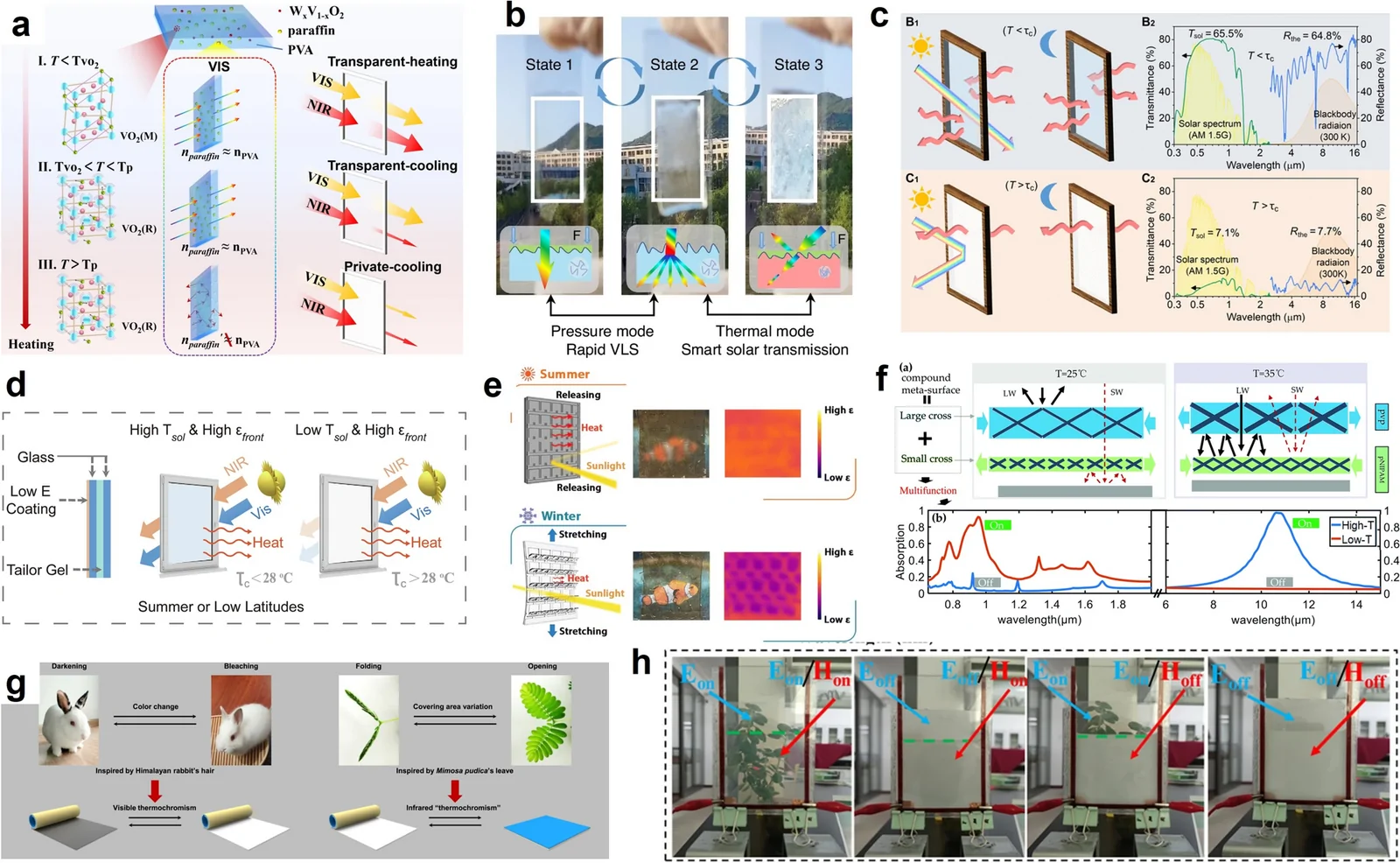
DRC materials based on multi-stimuli response structure. **a** The principle of temperature-adaptive tri-state smart window structure for W_*x*_V_1−*x*_O_2_/paraffin/PVA composite films [227]. Copyright 2024, Elsevier. **b** States of micropatterned thermochromic hydrogel under thermal and pressure modes [228]. Copyright 2024, Springer Nature. **c** Schematic of optical performance and the corresponding spectrum in hot (top) and cold (bottom) condition [229]. Copyright 2022, AAAS. **d** Scheme and usage scheme for the thermochromic smart windows [230]. Copyright 2023, Elsevier. **e** The working principle and photo of the durable solar/RC dual-control smart window in hot (top) and cold (bottom) condition [232]. Copyright 2023, Royal Society of Chemistry. **f** Design of both self-adaptive solar heating and radiative cooling with the compound cross metasurface [233]. Copyright 2020, Royal Society of Chemistry. **g** Design principle of the dual-mode thermal management device based on visible and infrared “thermochromism” [235]. Copyright 2022, National Academy of Sciences. **h** Photographs of composite windows in different states [237]. Copyright 2025, Springer Nature

Cao et al. [227] designed a tri-mode thermochromic composite thin film based on two PCM (W_*x*_V_1−*x*_O_2_ and paraffin), as illustrated in Fig. 12a. By utilizing different phase transition mechanisms, the system enables synchronized regulation in the solar and NIR spectrum. In the low-temperature zone, the transparent heating mode has a *T*_vis_ of 53.2%, which meets the requirement of indoor lighting and solar heating. In the mid-temperature zone, the metal–insulator transition of W_*x*_V_1−*x*_O_2_ activates a transparent cooling mode, maintaining high visible transmittance while significantly reducing near-infrared transmission (*T*_lum_ = 49.97% and Δ*T*_sol_ = 8.86%), thereby ensuring high visibility while minimizing cooling energy consumption. In the high-temperatures zone, the solid–liquid phase transition of paraffin induces a pronounced refractive index mismatch with the PVA substrate, resulting in intense light scattering, with a Δ*T*_sol_ of 33.7%. Inspired by squid skin, Wang et al. [228] developed a micropatterned thermochromic hydrogel based on pNIPAm, featuring two distinct optical regulation mechanisms: temperature-induced optical property regulation and pressure-controlled optical scattering. As depicted in Fig. 12b, the disruption and reformation of hydrogen bonds between polymer chains and water molecules across the phase transition enable a 61% modulation of visible light transmittance. Additionally, surface roughness variations under applied pressure facilitate a transition from diffuse to normal solar transmission. Huang et al. [229] integrated pNIPAm with silver nanowires to develop a smart window capable of regulating both solar transmission and thermal radiation (Fig. 12c). The temperature-triggered water capture and release associated enabled simultaneous solar and thermal regulation, achieving 58.4% solar control and 57.1% thermal regulation. Guo et al. [230] designed a hydrogel-based smart window exhibiting high transmittance, excellent tunable photothermal gain, and PRC properties (Fig. 12d). The pNIPAm hydrogel ensures superior solar light transmission and thermal gain, while a manual or mechanically reversible anisotropic glass template modulates the emittance. Long et al. [231] reported a reconfigurable interwoven surface capable of dynamically switching overlapping sequences to achieve spectral selectivity and ultra-broadband modulation (Δ*ε*_LWIR_ = 0.57). This approach enables windows, walls, and rooftops to exhibit tailored spectral tuning for enhanced energy efficiency.

Further extending this concept, Long et al. [232] proposed a dual-control smart window inspired by shape-morphing kirigami structures, designed for simultaneous solar transmission and PRC regulation. As shown in Fig. 12e, the strain-induced out-of-plane deformation of the origami structure exposes the underlying silver nanowires, enabling a long-wave infrared emittance modulation capacity of 0.5 through structural opening and closing control. Wang et al. [233] introduced a conceptual multilayer photonic architecture for temperature-adaptive solar and thermal radiation regulation. This structure, as shown in Fig. 12f, incorporating small and large cross resonators composed of silver arrays, enables tunable solar absorption and mid-infrared absorption. Ma et al. [234, 235], drawing inspiration from Himalayan rabbit fur and Mimosa pudica leaves, developed a dual-mode thermal management device utilizing shape memory polymers with selective electromagnetic spectral response. As illustrated in Fig. 12g, the integration of visible and infrared thermochromism enables autonomous temperature-driven switching between a heating mode (*α*_0.4–0.78_ = 73% and *ε*_LWIR_ = 0.28) and a cooling model (*R*_0.4–0.78_ = 65% and *ε*_LWIR_ = 0.95). Cao et al. [236] proposed a hybrid electrochromic–thermochromic structure combining PRC in the mid-infrared with maximized visible and near-infrared utilization. Utilizing a WO_3_/VO_2_ film structure with a controllable lithium-ion intercalation depth, the system enables three distinct active optical states, facilitating independent modulation of VIS and NIR transmittance. Yang et al. [237] combined electrochromism with thermochromism to create a Janus window based on a polymer-stabilized liquid–crystal film/thermochromic material. They employed an electrochromic layer as the primary control switch, while a thermochromic hydrogel layer served as an auxiliary functional module, achieving a combination of active and passive regulation. As shown in Fig. 12h, the smart window can achieve four distinct modes: highly transparent, electrochromic, thermochromic, and highly opaque. These functions are achieved through the synergistic effect between the electrochromic properties of the liquid–crystal layer and the thermochromic properties of the hydrogel layer. Table 3 summarizes a set of research categorized by the mechanisms mentioned above.

**Table 3:**
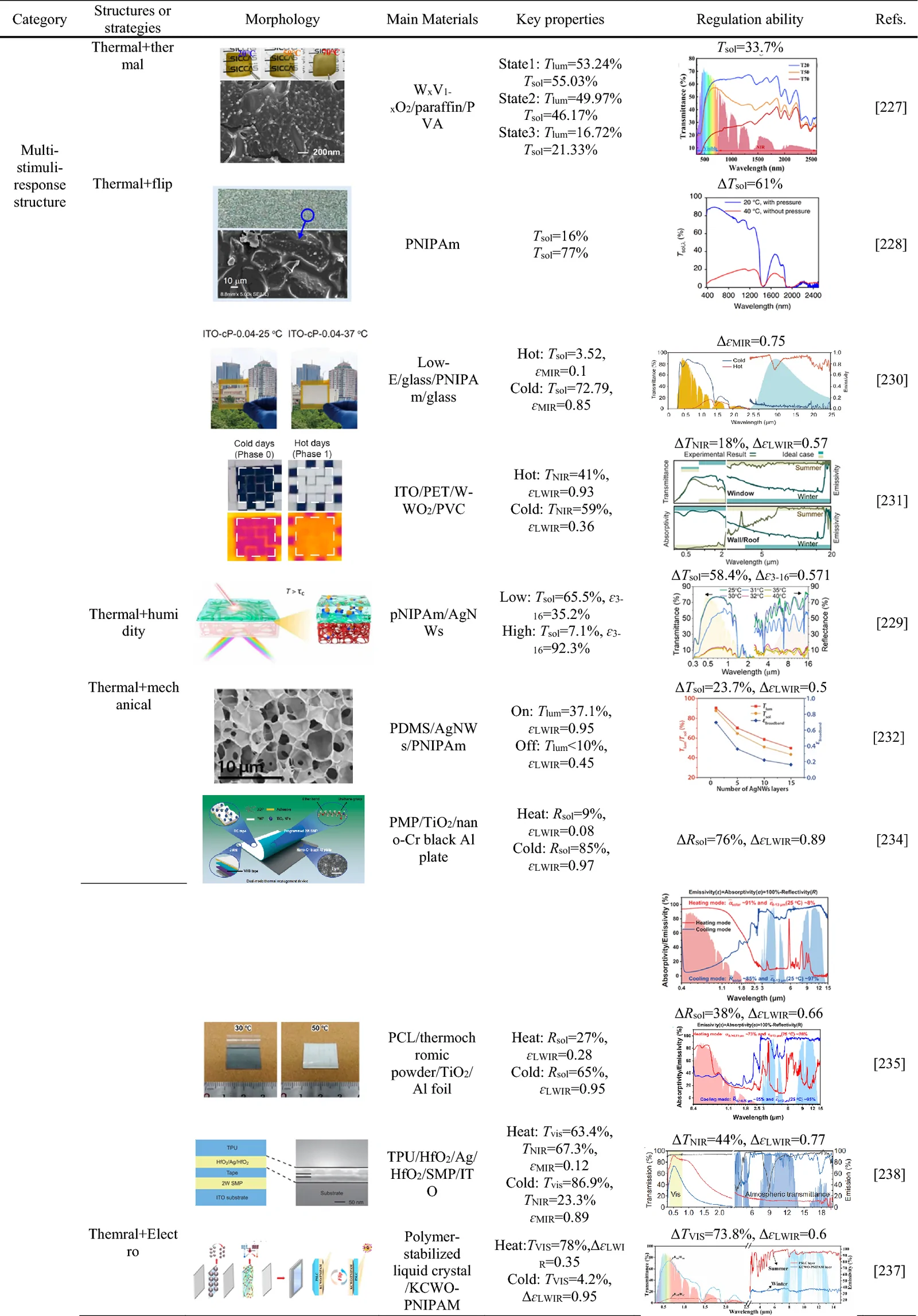
Summary of multi-stimuli response

## Challenges and Perspectives

**Fig. 13:**
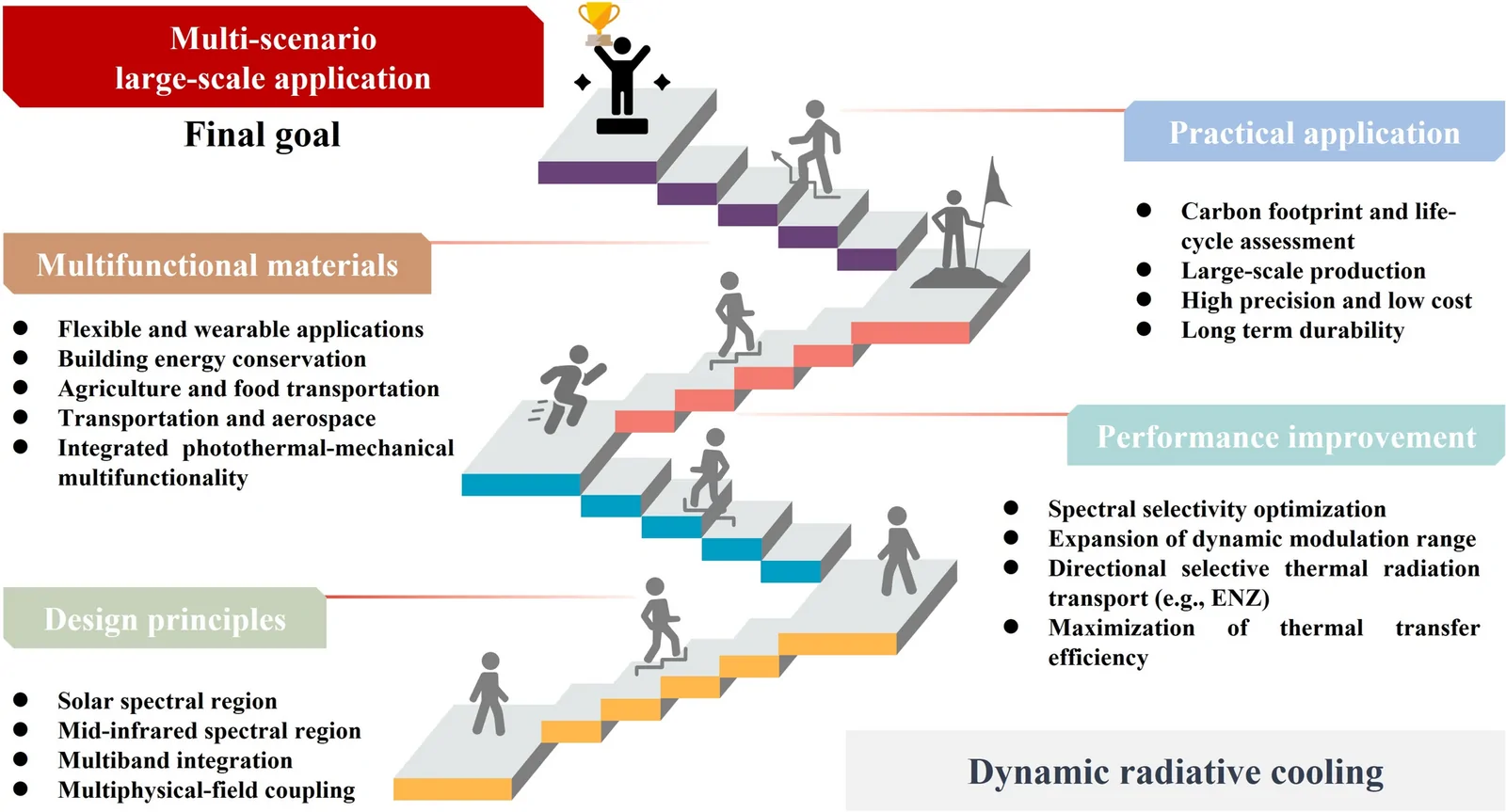
Practical challenges and prospects for large-scale applications of DRC

DRC technology, as an emerging radiative thermal regulation strategy, enables high-efficiency passive cooling through intelligent spectral regulation of materials under varying environmental conditions. This review provides a comprehensive summary of the latest advancements in DRC technology, covering its fundamental principles, intrinsic mechanisms, and various control strategies. Table 4 summarizes the main features on spectral modulation of these works, highlighting the challenges and issues in different structures. Despite significant theoretical and experimental advancements, large-scale commercial application of DRC remains fraught with challenges. As outlined in Fig. 13, key obstacles include the interfacial compatibility among different material components, the intricate interplay of multiple physical fields, and the optimal balance between response speed, regulation amplitude, and overall system efficiency. The suggested development perspectives are as follows:

(1) Development of novel multifunctional materials. Develop advanced material systems with both intelligent response characteristics and excellent radiative thermal regulation performance to further improve the environmental adaptability and expand the modulation range of materials. Beyond excellent thermal control, such materials are expected to exhibit multimodal triggering capabilities, enabling adaptive performance under diverse environments. For example, as mentioned in Sect. 3.2.3, magnetic response technology has excellent visible light control capabilities, but its potential as a DRC technology remains largely unexplored. At the same time, the introduction of biomimetic design (e.g., moth-eye structure and hierarchical structures) provides a new source of inspiration for material design [239]. Natural organisms, shaped by millions of years of evolution, exhibit exquisite control over light–matter interactions across multiple wavelength scales, and these strategies offer profound guidance for the development of DRC technology. Moreover, artificial intelligence technology plays an increasingly important role in the development of high-performance materials. By using machine learning, the exploration of compositional and structural of DRC materials can be quickly screened and optimized, enabling simultaneous optimization of both thermal and optical characteristics. In the recent work of Zhou et al. [240], the authors used a large number of AI technologies to precisely control the emission and reflection spectral characteristics of materials at the microstructure level, significantly broadening expanding the control range of PRC materials.

(2) Precision control in large-scale preparation. The main challenge for industrial application is to achieve large-area, high-precision, and cost-effective manufacturing. Particularly for materials such as electrochromic films, hydrogels, and other materials, which need to develop a more stable and efficient fabrication methods. These materials often have problems such as poor durability, limited uniformity of the extended area, and performance degradation during long-term operation, which seriously restrict their practical application [241]. Emerging continuous production techniques, such as roll-to-roll coating and inkjet printing, present viable solutions for large-scale functional material manufacturing. At the same time, the integration of bottom-up self-assembly strategies provides opportunities for achieving precise structural ordering at the micro- and nanoscale, thereby enhancing optical selectivity and radiation performance. Complementary to this, 3D printing provides unprecedented flexibility for manufacturing macrostructures with complex geometries [242]. The fusion of these methods not only enhances the scalability of advanced cooling materials, but also enables hierarchical structural design across multiple length scales.

(3) Seamless integration with thermal management systems. Practical applications necessitate compatibility with existing thermal management structure, especially in mature applications such as buildings, transportation, and electronic devices [243], are already highly optimized and deeply embedded within the operational framework. Consequently, the integration of DRC materials as isolated components often encounters practical barriers, since they cannot be directly interfaced with existing designs without extensive modification. On the other hand, replacing entire systems with novel DRC technology would demand high economic and technical costs, thereby limiting large-scale adoption. Recent research has shifted toward cross-scale system integration, such as integrating with photovoltaic and photothermal systems to improve efficiency, or integrating with temporary buildings to reduce energy consumption [244].

(4) Carbon footprint and life-cycle assessment. The development of environmentally friendly manufacturing processes has become a focal point of contemporary research. While life-cycle assessment (spanning cradle-to-grave evaluation from raw material acquisition, fabrication, deployment, to end-of-life treatment) is widely employed in industrial applications, a systematic approach remains still absent in the study of DRC technologies [245]. Current research focuses primarily on improving the optical properties of materials, but often overlooks the hidden environmental costs associated with large-scale manufacturing, including energy consumption, solvent use, greenhouse gas emissions, and the production of potentially harmful byproducts [246]. Furthermore, the sourcing of raw materials, especially transition metals and rare elements commonly used in electrochromic or nanostructured systems, can raise concerns about supply chain sustainability and ecological impacts. Researchers should evaluate energy efficiency and pollutant emissions across the entire material lifecycle.

**Table 4:** Summary of dynamic radiative cooling technology

Category	Strategies	Advantages	Limitations
Active response	Electro response	Fast response, precise regulation	Complex structure, high cost
	Mechanical response	Simple structure, good reversibility	Slow response, high-energy consumption
	Humidity response	Low cost, flexible regulation	Poor durability, difficult to apply
Passive response	Thermal response	No external energy, simple structure	Delayed response, limited control range
	Light response	Remote control	Poor stability, photodegradation
	Shape deformation	No external energy, strong adaptability	Limited deformation, poor stability
Multi-stimuli response	Thermal + filp	Simple and reliable	Complicated structure, poor durability
	Thermal + Mechanical	Strong regulatory ability	Delayed response, complicated structure
	Thermal + electro	Energy complementarity	Poor coordination

In conclusion, while PRC technology offers low-cost and maintenance-free heat dissipation, DRC technology provides adaptability by enabling radiative thermal modulation across varying environmental conditions. By integrating stimuli-responsive materials with PRC materials, DRC can overcome PRC’s inherent limitations of fixed optical properties and seasonal inefficiency. This adaptability positions DRC as a promising solution for next-generation smart thermal management in buildings, electronics, and human body.
